# Interfacial Self-Assembly of Sugars at Nanoscale Membranes Leads to Micron-Scale, Spectroscopically Ice-Like Chiral Suprastructures of Water

**DOI:** 10.1021/jacs.5c05215

**Published:** 2025-09-04

**Authors:** Li Zhang, Jinchan Liu, Kislon Voïtchovsky, Chaudhary E. Rani, Saranya Pullanchery, Jan Dedic, Victor S. Batista, Georg E. Fantner, Sylvie Roke

**Affiliations:** † Laboratory for Fundamental BioPhotonics (LBP), Institute of Bioengineering (IBI), School of Engineering (STI), 27218 École Polytechnique Fédérale de Lausanne (EPFL), Lausanne CH-1015, Switzerland; ‡ Department of Molecular Biophysics and Biochemistry, 5755 Yale University, New Haven CT06520, United States; § Physics Department, Durham University, Durham DH1 3LE, U.K.; ∥ Department of Chemistry, 5755 Yale University, New Haven CT06520, United States; ⊥ Laboratory for Bio and Nano Instrumentation (LBNI), Institute of Bioengineering, School of Engineering, 130376 École Polytechnique Fédérale de Lausanne (EPFL), Lausanne CH-1015, Switzerland; # Institute of Materials Science and Engineering (IMX), School of Engineering (STI), École Polytechnique Fédérale de Lausanne (EPFL), Lausanne CH-1015, Switzerland; ∇ Lausanne Centre for Ultrafast Science, École Polytechnique Fédérale de Lausanne (EPFL), Lausanne CH-1015, Switzerland

## Abstract

Life requires chemical chiral specificity. The emergence of enantioselectivity is unknown but has been linked to diverse scenarios for the origin of life, ranging from an extraterrestrial origin to polarization-induced effects, and magnetic field-induced mineral templating. These scenarios require an originating mechanism and a subsequent enhancement step, leading to widespread chiral specificity. The common denominator in all scenarios is water, which provides an environment for the enantioselective process. Because water is a nonchiral molecule, it has not been considered as an active ingredient in either of these processes. Here, we show that water can form extended chiral ordered structures that are induced by interactions with simple chiral prebiotic molecules, such as lipids and sugars. Using a combination of molecular dynamics simulations, chiral-sensitive and interface-specific vibrational sum frequency scattering, second harmonic scattering, and atomic force microscopy, the interfacial structure of water on nanoscale lipid membranes was investigated. Out-of-plane H-bonding interactions between achiral liposomes and simple chiral cyclic sugars lead to ordered, spectroscopically ice-like, chiral water suprastructures that extend along the self-assembled lipid-sugar complex over distances >10 μm. Such highly ordered self-assemblies could potentially have provided microenvironments that enable the enhancement of chirality.

## Graphical abstract

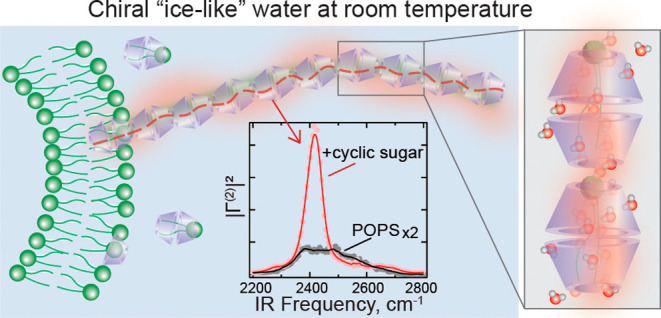

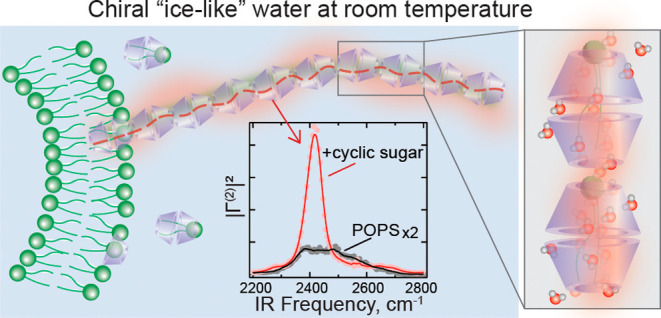

## Introduction

Chiral selectivity is one of the essential ingredients for the emergence of life on planet Earth.^1,2^ Sugars, amino acids, peptides and proteins, nucleotides, RNA and DNA are chiral and act in enantiospecific manners. For example, although DNA exists in two chemically identical mirror image forms called enantiomers, only right-handed (D) DNA participates in life’s chemical transformations. Understanding the reasons behind this selectivity is an important question that links back to the origin of life.^3,4^ The synthesis of proteins (mediated by DNA and RNA), as well as the replication of DNA, works only when the polymeric chains are made of enantiomerically pure building blocks, as H-bonding patterns are disrupted upon the introduction of mirror-image impurities.^5,6^ How this chiral specificity was created in the early stages of planet Earth is an ongoing debate, reviewed in refs. 3,4 To achieve biological chiral specificity, a chiral bias is required, followed by amplification/enhancement. The chiral bias has been hypothesized to originate from the inherent properties of matter, the interaction of fields with matter, or the interactions between the chiral molecules themselves, among other sources. The amplification process has been suggested to be either terrestrial or extra-terrestrial in origin, involving possibly stereoselective polymerization, asymmetric catalysis, or self-disproportionation of enantiomers.^4^ For example, recently, a combination of spin-selective crystallization of an RNA precursor (racemic ribo-aminooxazoline) on a magnetite surface achieved a significant enantiomeric excess, which was increased to 100% selectivity upon repeated crystallization.^7^ In this scenario, it is hypothesized that a shallow-lake environment on early Earth, in combination with magnetite, would be needed to create enantioselectivity.

Interestingly, the key ingredient in all these scenarios, which is always present but not explicitly included in discussions, is water. Hydration is known to be essential for the evolution and equilibration of the structure of (bio)­molecules,^8,9^ and it can impart important structural biases on biological systems. An example is the charge of most biological membrane interfaces, which is negative but not positive. Even though a mean-field dielectric representation of water predicts that there is no difference between the hydration of positive or negative ions with equal charge and size,^10^ nature has evolved with a predominantly negative surface charge. This choice seems arbitrary. However, recent molecular level measurements have shown that a ∼ 2*k*_B_ *T* free energy penalty for hydrating cations versus anions exists in aqueous solution,^11^ which arises from charge-asymmetric H-bonding interactions that are also present at aqueous lipid membrane interfaces.^12^ This difference in energetics is inherent to the molecular structure of water and may have steered lipid cell membranes into being primarily negatively charged. It is, therefore, of great interest and relevance to ask similar questions about the amplification process that is needed to create a widespread chiral specificity, essential for the origin of life.

Nonchiral molecules, such as water, can adopt a chiral structure induced by interactions with chiral biomolecules. Chiral- and interface-specific vibrational sum frequency generation (SFG, see e.g., ref. 13–17 ) measurements have shown that surface-immobilized DNA,^18,19^ artificial aquaporins,^20^ and peptides^21,22^ can induce a chiral superstructure of water. The extent over which chiral water was templated by such biomolecules could not be estimated from the reflection-mode SFG data, and molecular dynamics (MD) simulations^23^ concluded that chiral water in these systems is only located at the first hydration layer around the biomolecules that extends perpendicularly into the aqueous phase. The sizes of such chiral superstructures are limited by the method in which they are obtained, with the biomolecules being drop-casted on a planar extended surface. Because of this, and the limited number of systems studied (DNA and peptides), it is not well understood yet if simple chiral molecules, which were likely to have been present under prebiotic conditions, can self-assemble with water into more extended chiral structures that exceed over microns.

Here, we address the question if water can form chiral suprastructures via chiral sugar complexation in solution and investigate if spontaneous interfacial complexation and subsequent molecular self-assembly can result in extended chiral structural transformations. To do so, we investigated the structure of water inside chiral cyclodextrin complexes using MD simulations and found that self-assembled pairs of methylated-β-cyclodextrin (mβCD) contain chiral water. Experimental studies using dynamic light scattering (DLS) and second harmonic scattering (SHS) showed that, in the presence of phosphatidylserine liposomes, extended chiral structures were formed and estimated to be up to 10 μm long. Vibrational sum frequency scattering (SFS) experiments from the surface of liposomes showed that these structures contain spectroscopically ice-like water at room temperature, which is highly ordered and chiral. Such structures can only be formed through an out-of-plane arrangement of primarily intermolecular H-bonding interactions, along the direction of the symmetry axis of the self-assembled complex. Since these building blocks for forming extended chiral structures of water were likely present under prebiotic conditions, the formation of extended chiral structures may have been of relevance to the emergence of enantiospecificity.

## Results and Discussion

### Can Water Form Chiral Structures within Self-Assembled Cyclic Sugar Dimers?

Cyclodextrins (CDs) are chiral cyclic glucose complexes that serve as an ideal starting point for our investigation into extended chirality, because they form water-soluble inclusion complexes with water and guest molecules such as lipids.^24–26^ mβCD is a prime example, composed of 7 d-glucose molecules linked by α(1–4) bonds, forming a truncated cone shape (Figure 1A, highlighted in blue), with the wider and the narrower rim referred to as the secondary and primary rim, respectively. On average, 1.7–1.9 of the 3 hydroxyl groups of mβCD are methylated, which significantly increases its solubility in water (≥500 mg/mL vs ∼ 18.5 mg/mL)^27^ compared to native βCD having more intramolecular H-bonding.^28,29^ mβCD is chiral and because out-of-plane interactions can occur, we expect that this chirality is transferred onto achiral inclusions.^14,30^ To determine if this is possible, we start by investigating mβCD dimers in water with classical MD simulations.

**Figure 1:**
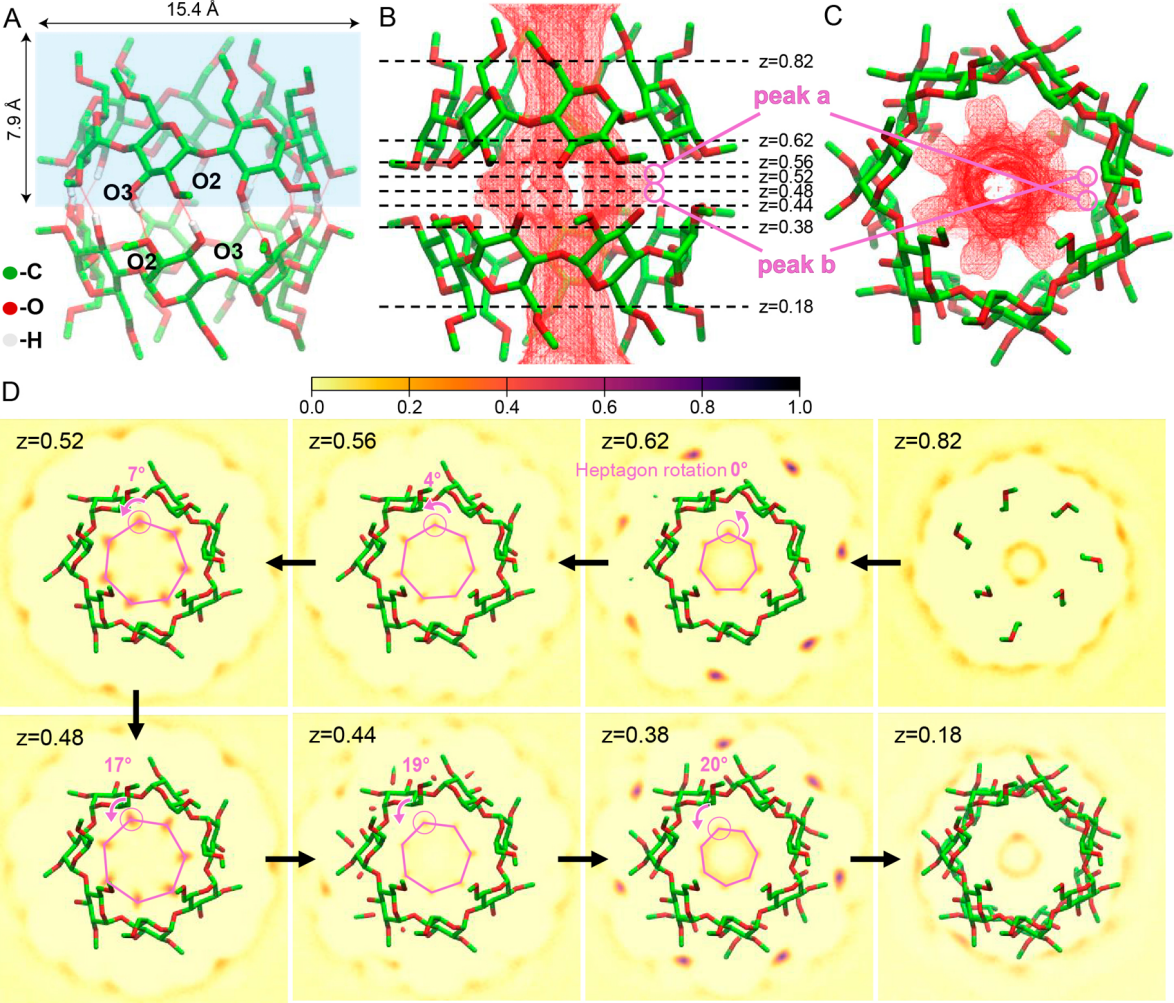
Chiral water within the cage of mβCD dimer. A: Stable mβCD dimer formed by two mβCD monomers with their secondary rims facing each other. The methylated O2 groups and the O3 groups are indicated. The mβCD monomer is indicated by a blue box. B: Water density distribution within the mβCD dimer. Dashed lines identify the parallel planes at different z coordinates. Peak a and b are the water density peaks near the O2 and O3 groups at the interface. C: Cross-sectional view of the water density. D: Water density in slices perpendicular to the *z*-axis from z = 0.82 to z = 0.18. Its value is represented by color, with darker color indicating higher density, as shown in the color bar. The pink heptagon outlines the water density peaks inside the mβCD cavity, which aligns with the heptagon ring of mβCD at z = 0.62 and rotates incrementally from 0° (z = 0.62) to 20° (z = 0.38), indicated by pink arrows. See Video S1 for details of all slices.

The MD simulations were performed using NAMD^31^ and the CHARMM36 force field^32^ as described in the SI, S1. The results are summarized in Figure 1. Two mβCD monomers can form a stable mβCD dimer in vacuum with the secondary rims facing each other (see Figure 1A). This configuration is stabilized by 14 intermolecular H-bonds formed between the methylated O2 groups (acting as H-bond acceptors) and the O3 groups (H-bond donors) (indicated in Figure 1A). Inserting the dimer in a (TIP3P^33^) water box results in water-dimer complexation. The calculated water density within the mβCD dimer is shown in Figure 1B-D. The obtained water density distribution in Figure 1B and the cross-sectional view in Figure 1C show that water within the dimer cage is highly ordered and presents a centrosymmetric heptagonal pattern that aligns with the mβCD geometry. Figure 1D shows the water density in slices perpendicular to the *z*-axis. Between z = 0.62 and z = 0.38 (Figure 1B), the heptagonal water peaks rotate incrementally from 0° to 20° (arrows in Figure 1D) with respect to the heptagon ring of mβCD, showing a spiral arrangement of water (see Video S1). This spiral structure is chiral and thus demonstrates the possibility of hydrating water molecules within the mβCD internal cavity to assemble into chiral suprastructures, which is likely driven by intermolecular H-bonding. Larger complexes formed by interactions between more than two mβCD rings might lead to extended chiral arrangements of water. Within such arrangements, there would be a single layer of water molecules in a ring-like structure (Figure 1), which extends along the direction of the stacked multimer. Next, we investigate this possibility experimentally.

### Chiral Water Suprastructure Extends over Several Microns

SHS is a nonlinear light scattering experiment in which a femtosecond near-infrared (NIR) laser beam interacts with a solution and emits second harmonic (SH) radiation. This nondirectional incoherent light scattering occurs for every molecule that has an anisotropic electronic structure. This response is very weak, but it is emitted from nearly every material.^34,35^ When anisotropic molecules are organized in such a way that they experience orientational cross-correlations, a stronger coherent contribution appears in the emitted intensity. SHS also has a particular sensitivity to chirality, which can be recorded using specific polarization combinations.^36,37^ Bulk water has been shown to emit both coherent and incoherent contributions, but only in the achiral polarization combinations.^38^ To measure the effect of mβCD on bulk water, we recorded SH emission at different scattering angles *θ*, and polarization combinations. Nonchiral emission occurs with all beams polarized in the horizontal scattering plane (PPP), or the vertical scattering plane (SSS). SSS probes nonchiral incoherent emission only, which emerges as a constant intensity for neat water, and is therefore useful for normalization purposes. In a nonresonant SHS experiment, water generates the vast majority of SH response, as the intensity scales with the molecular number density squared.^39^ Chiral structures emit with the SH beam polarized perpendicular to the scattering plane and the incoming beams parallel to it (SPP). Therefore, we plot $S{\left(\theta \right)}_{\mathrm{XPP}}=\left(I{\left(\theta \right)}_{\mathrm{sample}}^{\mathrm{XPP}}-I{\left(\theta \right)}_{\mathrm{solvent}}^{\mathrm{XPP}}\right)/I{\left(\theta \right)}_{\mathrm{water}}^{\mathrm{SSS}}$ , with X = P for nonchiral and X = S for chiral structures. Details of this method can be found in SI, S1 and S2. Figure 2A shows the recorded SHS patterns from a bulk solution of 1 mM mβCD in water (D_2_O) with 25 mM NaCl in PPP (black, nonchiral) and SPP (blue, chiral) polarization combinations. Figure 2A shows no detectable SHS response from the mβCD solution. This means that mβCD does not significantly change the water structure, or it does so in a way that it is below the detection limit. For a chiral isotropic bulk medium, the nonchiral elements of the second-order susceptibility tensor vanish and the orientational averaging significantly reduces the chiral susceptibility.^15,40,41^ Moreover, the degenerate nature of two input beams significantly reduces the chiral output^40–42^ to such a degree that it would likely not be detectable even if it was present.

**Figure 2:**
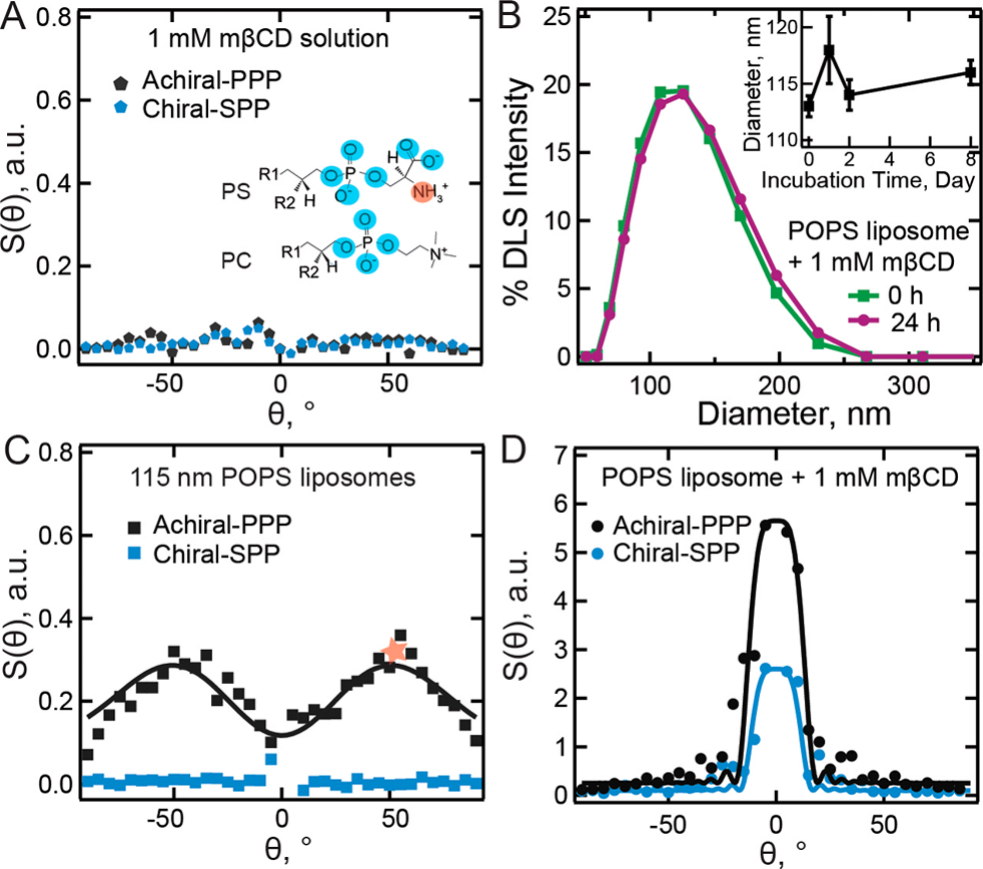
Chiral interfacial water extends over several microns. A: The achiral (PPP, black star) and chiral (SPP, blue star) SHS patterns of 1 mM mβCD solution in water. The inset shows the structures of phosphatidylserine (PS) and phosphocholine (PC) lipid headgroups, where the possible H-bond donors (acceptors) are indicated in red (blue) circles. B: DLS size distributions of POPS liposomes before (green, 0 h) and after (purple, 24 h) 24-h incubation with 1 mM mβCD. The inset shows the average POPS diameter for different incubation times. C: Achiral (PPP, black square) and chiral (SPP, blue square) SHS patterns of POPS liposomes in water. The black solid line is the scattering pattern computed using nonlinear light scattering theory for spherical particles (SI, S2). The star indicates the angle of maximum intensity at which the SFS data in Figure 3 was recorded. D: Achiral (PPP, black dot) and chiral (SPP, blue dot) SHS patterns of POPS liposomes after 24-h incubation with 1 mM mβCD. The solid lines are the form factor curves computed using nonlinear light scattering theory for cylindrical particles (explained in the SI, S2). All solutions contained 25 mM NaCl.

Therefore, to observe chiral suprastructures generated by the self-assembly of mβCD, a higher degree of anisotropy needs to be present, for example, by additional interfacial interactions. We thus added liposomes to the solution. Liposomes are spherical vesicles made out of lipid bilayers, and they were also likely present during the prebiotic stages of planet Earth.^43^ mβCD interacts and self-assembles with lipids and liposomes^44,45^ in various applications. Cyclodextrins can assemble into supramolecular structures through intermolecular H-bonding or host–guest molecular interactions.^46^ In order to generate chiral suprastructures by means of lipid-sugar interactions, out-of-plane interactions are needed. A lipid that has multiple possible H-bond interactions, such as, 1-palmitoyl-2-oleoyl-*sn*-glycero-3-phospho-l-serine lipid (POPS) meets this requirement with 3 types of H-bonding sites, whereas a lipid with a phosphocholine (PC) headgroup does not. The H-bond donating (blue) and accepting (red) sites of PC and PS headgroups are illustrated in Figure 2A.

Liposomes were prepared by extrusion as described in the Methods Section S1 and characterized by DLS. The liposome dispersions were then incubated with 1 mM mβCD solution for a period of 24 h or longer. Figure 2B shows the diameter distribution of POPS liposomes after 0 h (green) or 24 h (purple) incubation. The average diameter is ∼ 115 nm, and it is not affected by the incubation with mβCD for over 8 days (Figure 2B inset). SHS patterns of neat POPS liposomes are shown in Figure 2C. The achiral response (black) shows the characteristic two-lobed SHS pattern of charged liposomes (see e.g., refs. 12,47 ) which is well described by the theory of nonlinear light scattering from spherical shells (see SI, S2 and S3). The chiral response (blue) is absent in an aqueous solution of liposomes, likely due to their overall nonchiral spherical morphology, despite the chiral center on the middle glycerol carbon.

Having observed that chiral water is not detected at the interface of liposomes nor in mβCD solution, we next investigate a mixed dispersion of liposomes and mβCD. POPS liposomes were incubated with 1 mM mβCD for 24 h to ensure sufficient interaction time. Figure 2D shows the recorded achiral (black) and chiral (blue) SHS patterns. Surprisingly, both the patterns and their intensities have changed. The achiral response has acquired a forward-centered peak, and the intensity at the angle of maximum intensity has increased from ∼ 0.2 x to ∼ 5.8 x the SSS intensity of neat water. The chiral response, which is absent for neat liposomes, has a pattern similar to the nonchiral one with comparable intensity. Such a sharply peaked forward-centered scattering pattern as in Figure 2D cannot be obtained from spherical shells.^37^ Thus, the shape of the object giving rise to this prominent feature of the SHS pattern is likely not spherical. Furthermore, the width of the SHS patterns is indicative of the size of the scattering object, with a narrow scattering pattern arising from a large object and a wide scattering pattern arising from a small object, compared to the wavelength of the emitted light. Thus, the object that gives rise to the features in Figure 2D has become much larger than ∼ 115 nm.^48,49^ Interestingly, the DLS shows no difference before and after incubation.

This apparent contradiction between DLS and SHS can be explained by the different contrast mechanisms. Linear light scattering (DLS) is determined by the refractive index contrast between the inside and the outside of an object and scales with the square of the change in refractive index multiplied by the object’s volume. With this contrast mechanism, bulk amounts in the μM range and above can typically be detected.^50^ For nonresonant SHS, the contrast arises from orientational correlations between anisotropic molecules and is thus exquisitely sensitive to changes at interfaces^51,52^ or other ordered structures.^11,53,54^ The detection limit of SHS is down to the picomolar range, and a single protein adsorbed to a liposome in solution can be detected, while it is invisible for DLS.^55^ The data in Figure 2B,D can collectively be explained if the SHS arises from a long structure with a chiral structure and a small volume, such as a thin flexible rod with a width of ∼ 1 nm, on the order of magnitude of the width of an mβCD molecule. Such rods emit forward-peaked SHS patterns in both chiral and achiral detection channels. Using nonlinear light scattering theory, it is possible to compute the SHS pattern with only the form factor expression (eq S14 in SI, S3). Note that this is likely not the precise pattern since the values of the susceptibility tensor elements are unknown and difficult to be estimated in this case. Nevertheless, the form factor alone provides a good qualitative indication of the pattern shape, as well as the approximate dimensions of the object (see ref.^56^). The solid black (achiral) and blue (chiral) lines in Figure 2D are computed SHS patterns from a ∼ 1 nm wide and ∼ 10 μm long cylinder. The SI (S2 and S3) contains the full computation. Such a cylinder is potentially composed of many stacked cyclodextrins, which likely contain hydrating water inside. Because of the very small volume of this thin cylinder, it is most likely not detected in a DLS experiment.

SHS experiments on liposomes thus show in Figure 2 that a strong chiral SH response emerges when POPS liposomes are incubated with mβCD. It exhibits a pattern that arises from a long, thin, and flexible rod growing away from the liposome interface into solution. Since SHS generally reports on water, the chiral response likely emerges from water organized in a chiral supra-structure that could be several microns long and protrudes from the surface of POPS liposomes. All these experiments were also performed with POPC liposomes (shown in Figures S3 and S5). In this case, the SHS patterns do not display changes when mβCD is added to the POPC liposome solution, indicating that an out-of-plane H-bonding interaction is essential. To obtain more information about the molecular nature of the observed chiral suprastructures, vibrational SFS spectra were recorded.

### Interfacial Chiral Water Becomes Spectroscopically Ice-Like

In a vibrational SFS experiment (sketched in Figure 3A), two laser beams with IR and visible (VIS) frequency are spatially and temporally overlapped inside a solution containing liposomes and mβCD. The scattered sum frequency (SF) beam is collected at an angular range of *θ*=55° ± 20°, which corresponds to the angle where the maximum intensity is emitted (indicated by the red star in Figure 2C). SFS involves a simultaneous IR and Raman transition (Figure 3A), which is highly interface-selective due to the symmetry selection rules. The frequency distribution and intensity of the scattered SF beam probes anisotropic orientational distributions of vibrational modes at interfaces. Using S-polarized SF and VIS light (i.e., the waves oscillate perpendicular to the scattering plane) in combination with P-polarized IR light (i.e., the waves oscillate parallel to the scattering plane) (SSP), achiral interfacial molecular groups are measured. Chiral interfacial groups are probed with the PSP (or PPS, or SPP) polarization combination.^37^

**Figure 3:**
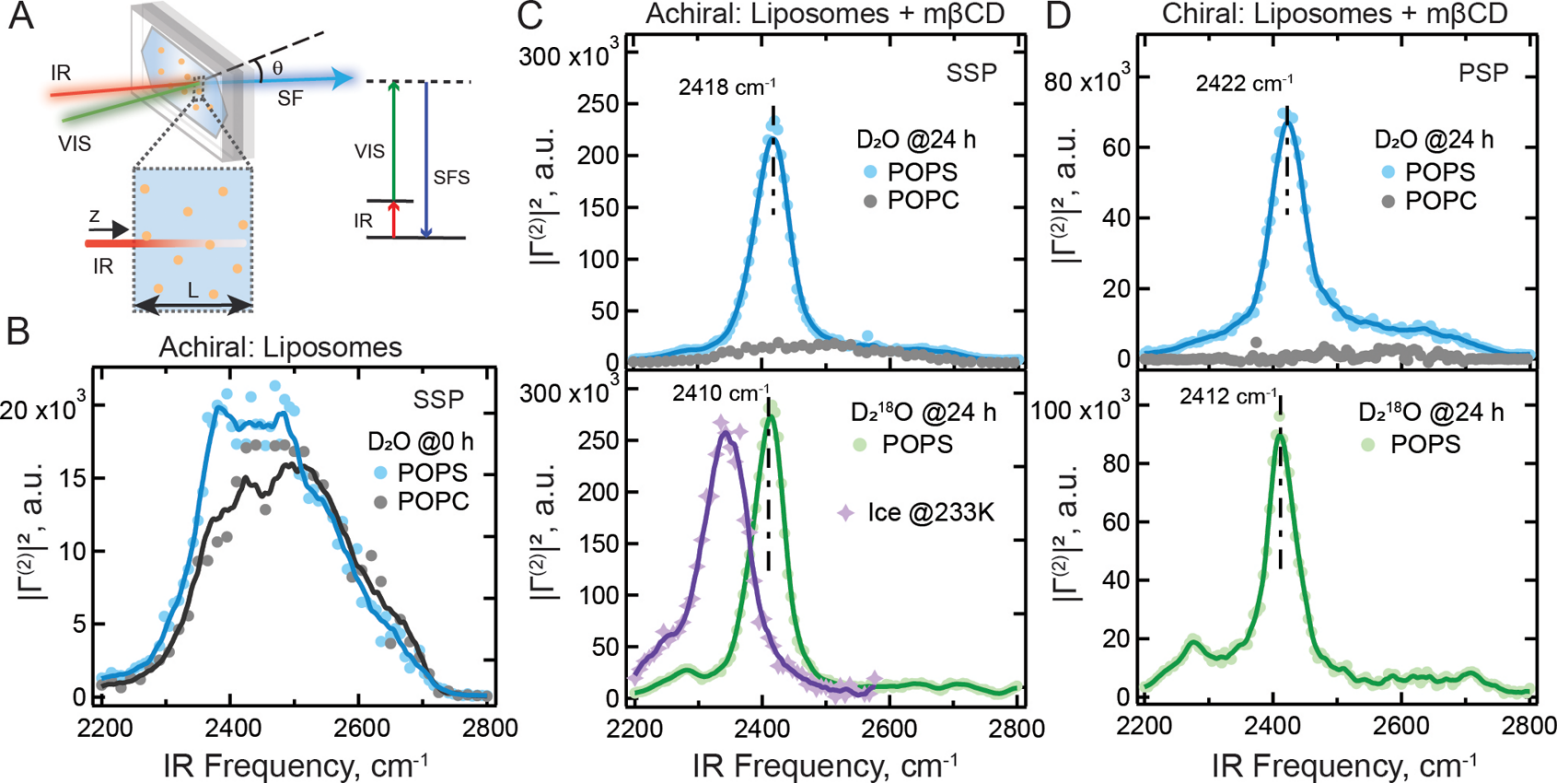
Spectroscopically ice-like chiral water at room temperature. A: Sketch of the vibrational SFS experiment and the corresponding energy level diagram. B: Vibrational SFS spectra in the O–D stretch region of POPS (blue) and POPC (black) liposomes in a heavy water solution containing 25 mM NaCl measured using the SSP polarization combination. C: Achiral vibrational SFS spectra in the O–D stretch region of POPS (blue) and POPC (black) liposomes in D_2_O (top) and POPS liposomes in D_2_^18^O (green, bottom) after 24 h of incubation with 1 mM mβCD. The SFS spectrum of ice nanocrystals measured at 233 K from ref.^57^ is also shown (purple) for comparison. The spectra were recorded using the SSP polarization combination. D: Chiral vibrational SFS spectra in the O–D stretch region of POPS (blue) and POPC (black) liposomes in D_2_O (top) and POPS liposomes in D_2_^18^O (green, bottom) after 24 h of incubation with 1 mM mβCD. The spectra were recorded using the PSP polarization combination. All solutions contained 25 mM NaCl. The solid lines in B-D are running averages provided as guides to the eye.

The SFS experiments were conducted using heavy water (D_2_O), which behaves the same as H_2_O in the OH/OD stretch region,^58^ considering the benefits of laser performance in the O–D stretch range. The SFS response in the 2200–2800 cm^–1^ frequency region reports on the vibrational O–D stretch modes of interfacial water, which reveals the structure of the H-bond network at the interface. The SF intensity spectrum is significantly modified by IR absorption that arises as the IR beam propagates through the bulk aqueous solution. This absorption can be removed from the data via a procedure (described in SI, S1), which generates the actual liposome surface response^59,60^ given by the effective second-order particle susceptibility ${|\Gamma^{\left(2\right)}|}^{2}$ . Figure 3B shows the obtained achiral SFS ${|\Gamma^{\left(2\right)}|}^{2}$ spectra in the O–D stretch region for POPS (blue) and POPC (black) liposomes. Before incubation (at 0 h), POPS liposomes generate achiral water responses and the resulting achiral SSP spectrum in Figure 3B (blue) reflects the broad frequency range of the O–D stretch modes. It comprises a broad peak from 2300 to 2700 cm^–1^, with a maximum intensity from 2375 to 2500 cm^–1^, indicating the presence of various H-bonding environments. The POPC spectrum (Figure 3B, black) has similar spectral features. These achiral responses are expected as the interfacial water on the inner and outer leaflets adopts different structures, which are primarily dictated by the difference in the electric double layer between the inner and outer liposome leaflets.^61^ The spectral shape is similar to the reflection mode-SFG spectrum of a lipid monolayer, composed of PS headgroup, on the air/water interface^62–64^ and the SFS spectrum from oil droplets.^60^ Meanwhile, no visible chiral spectral signature is detected from achiral liposome interfaces in Figures S4 and S6, consistent with the absence of chiral SHS intensity (Figure 2C, blue). The chiral responses emerge from POPS liposomes after ∼ 1-h incubation with 1 mM mβCD and continue to increase over time, but not for POPC liposomes (see Figures S4 and S6C). Figure 3C (top) shows achiral SFS ${|\Gamma^{\left(2\right)}|}^{2}$ spectra in the O–D stretch region for POPS (blue) and POPC (black) liposomes after 24-h of incubation with 1 mM mβCD in D_2_O, and Figure 3D (top) shows the respective chiral responses. After sufficient incubation (∼24 h), both achiral and chiral spectra from POPS liposomes have changed drastically: The intensity of the achiral response has increased by a factor of ∼ 10 in Figure 3C (top, blue). Additionally, a narrow peak centered at 2418 cm^–1^, 50 cm^–1^ in width (full width at half-maximum, fwhm) has appeared on top of the original broad spectral feature. For the chiral interfacial water, a similar peak centered at 2422 cm^–1^ appeared in the spectrum (Figure 3D top, blue).

This narrow spectral feature in the center of the vibrational spectrum of water is surprising. There are three present molecules that can possibly provide such a peak: N–H (or N-D) vibrations from the lipids, highly structured O–D groups from the mβCD molecules and D_2_O. In what follows we will consider these options in detail. Chiral reflection mode SFG experiments of peptides in contact with H_2_O^22,23^ show a narrow peak (fwhm ∼ 70 cm^–1^) at 3270 cm^–1^, on top of a broad signature. In this case, the β-sheet peptide contains many N–H groups that give rise to this sharp spectral feature as revealed by isotope dilution of water. Based on gas phase ratios,^65^ an N–H stretch mode at 3270 cm^–1^ corresponds to N-D stretches in the range 2515–2319 cm^–1^, which overlaps with the vibrational frequency of the O–D stretches from water. Since each PS headgroup contains one -NH_3_^+^ group, this could potentially be responsible for the sharp SFS response around 2420 cm^–1^. It should be noted that POPS in this study has only a single N-D group, instead of a repetitive N-D structure as was the case in the peptide study of ref.^21^ Given the total number of N-D groups in the liposome system, the drastic changes in the SHS intensity of Figure 2D do not likely emerge from nonresonant N–H responses. Nevertheless, to unambiguously identify the spectral contributor, we performed SFS experiments using double heavy water, D_2_^18^O, as the solvent. According to previous studies, replacing D_2_O by D_2_^18^O shifts the O–D stretch modes by ∼ 10 cm^–1^ to lower frequency.^66^ N-D modes are not shifted in frequency by this isotope substitution, and neither are the hydroxyl groups of the mβCD molecule. Furthermore, the stacked mβCD molecules, shown as a dimer structure in Figure 1, have oppositely oriented hydroxyl groups, which renders their O–D vibrations SFS inactive.

The recorded SFS responses upon the isotope substitution are shown in the bottom panels of Figure 3C,D (green traces). The achiral and chiral SFS spectra from the 24-h incubated mβCD-liposome dispersion in D_2_^18^O have central peaks at 2410 and 2412 cm^–1^, shifted down in center frequency by ∼ 8 cm^–1^ (achiral, Figure 3C, green) and ∼ 10 cm^–1^ (chiral, Figure 3D, green), respectively. Figure S7 and Table S7, Section S4, contain detailed global fits to the spectra. The observed spectral shifts are therefore close to the reported frequency shifts in vibrational O–D stretches to^18^O–D stretches upon isotope substitution.^66,67^ We thus conclude that the prominent peaks in the SFS spectra in Figure 3 after mβCD incubation originate from water molecules.

A spectrally narrow water peak is reminiscent of the SFG spectrum of ice nanoparticles,^57^ and of planar extended ice surfaces.^68,69^ To illustrate this more clearly, an SFS spectrum of 100 nm solid D_2_O nanoparticles (recorded at 233 K^57^ has been added to Figure 3C (bottom, purple) for comparison. The sharply peaked water signature is comparable in shape to the ice nanocrystal peak. The difference is that the width of the peak in the blue spectrum (top, Figure 3C) is narrower, and the center frequency is shifted to higher frequency (∼ 65 cm^–1^). The IR and Raman spectra of ice lend their characteristic spectral shape from molecular couplings of ordered molecules,^70^ which is also reflected in the SFG spectrum of ice. Intermolecular coupling broadens the SF spectrum of water, and a higher temperature moves the central peak to higher frequency. We can estimate how the temperature influences the central peak frequency by examining SFG spectra of planar basal ice.^71^ A spectral shift of ∼ 0.7 cm^–1^/K^71^ can be expected for D_2_O. If the nanocrystal SFS spectrum had been taken at room temperature but not at 233 K, the central frequency of the ice nanocrystals would have shifted from ∼ 2350 cm^–1^ to ∼ 2400 cm^–1^, which is quite close to the measured value. Since the central frequency is correlated to the distance between the two O atoms of neighboring water molecules,^70^ this frequency shift indicates that the O–O distance between two adjacent water molecules has increased by ∼ 0.05 Å. Therefore, based on the isotope dilution experiment and the comparison to known spectral features of ice, we conclude that the sharply peaked and intense spectra originate from water molecules that form an ordered structure in which the spacing of water is a bit larger than in 273 K ice.

Thus, combining the data in Figures 2 and 3, we find that the interaction of cyclic chiral sugars with water-membrane interfaces leads to a chiral, highly ordered structure that extends in the direction of the flexible cylinder, and exists at room temperature. The MD simulations suggest that this is imparted by the inner ring structure of self-assembled cyclodextrins.

### Self-Assembly of mβCD-POPS-Water Complexes

Figures 2 and 3 show that interfacial water is highly perturbed by the presence of cyclodextrin, forming extended ordered chiral arrangements. It is expected that mβCD complexation with POPS lipids on liposomes is an important prerequisite for the formation of such structures. The structure of POPS lipids in these interfacial complexes were also studied. We measured the vibrational SFS spectra of the P–O stretch modes from lipid headgroups (Figures S4 and S6A) and the C–H stretch modes from lipid chains and mβCD (Figures S4 and S6B), for both POPS and POPC liposome solutions. Figure S6C shows the integrated SFS response over time. Initially, no detectable SFS modes were present before incubation as the lipids are distributed equally across the inner and outer leaflets, leading to symmetric configurations that thus are SFS inactive.^61^ The detected SFS response from POPC liposomes remains absent after 24-h incubation with mβCD, and we conclude that no interfacial complexation occurs. However, POPS liposomes show drastic changes. The SF intensity of the P–O and the O–CO groups increases drastically in the achiral as well as the chiral responses. This suggests that the lipid headgroups adopt a chiral arrangement. A similar behavior is seen in the C–H stretch region (Figure S6B) that reports on both the cyclodextrins and the lipids.

This surface selectivity is likely determined by intermolecular H-bonding, as suggested by the MD simulations in Figure 1. Two mβCD monomers form a stable dimer in Figure 1A via intermolecular H-bonds between their O2 groups (H-bond acceptor) and O3 groups (H-bond donor). PS lipid headgroups exhibit 3 H-bonding sites with both possible H-bond donors and acceptors, which are marked as red and blue circles, respectively, in the inset of Figure 2A. This lipid structure facilitates intermolecular H-bonding between neighboring POPS, mβCD, and water molecules, in an out-of-plane fashion, to create chiral self-assembled suprastructures. In comparison, the PC headgroup (Figure 2A inset) has only 1 H-bond sites (H-bond acceptors), which thus makes it less favorable for intermolecular interactions, and impossible to create a chiral assembly.

The SHS experiments could be explained with a long, thin, flexible rod-like mβCD-lipid–water complex that grows out of the liposome interface. The SFS experiments unequivocally demonstrated the presence of highly ordered chiral water. Figure S6 revealed that lipids also become asymmetric and chiral. Based on these observations, we suggest a mechanism for the interfacial self-assembly of water–lipid-mβCD complexes that leads to such structures (illustrated in Figure 4A). When mβCD is added to the solution of liposomes, some adsorb on the membrane surface (i). The adsorbed mβCD molecules then diffuse along the surface (ii). POPS lipids in the outer leaflet can interact with cyclodextrins and can become encapsulated within the cavity of mβCD together with hydrating water via intermolecular H-bonding, as well as the hydrophobic interactions between the lipid tails and the hydrophobic mβCD cavity. The formed mβCD-lipid–water complexes can also diffuse along the membrane or leave the membrane surface (iii). Since the DLS measurement did not detect significant changes, the dissolution is unlikely to occur frequently. With increased incubation time, some mβCD-lipid–water complexes can assemble to form longer structures which protrude out of the membrane interface (iv). Many such interactions might occur and lead to diverse structures. A tiny subpopulation of all these structures contains an assembly of many cyclodextrin and water molecules, growing into structures up to several microns long. Since self-assembly of surfactants, lipids, and cyclodextrins can grow into extended multimicrons long structures,^72,73^ the proposed mechanism is feasible and is strongly supported by the simulations and experimental scattering data presented here. We note that it cannot be excluded that the strong SFS and SHS responses may arise from microcrystals of mβCD that first nucleated on the liposome surfaces and then detached. This would require an additional detachment step beyond the mechanism illustrated in Figure 4. We do not expect this scenario to be the main source for our experimental observations, since the mβCD concentration used here is below its solubility limit in aqueous media (1 mM vs 38 mM), and the intensity growth curve of the SFS response (Figure S6C) is monotonous. Control experiments using a lipid solution instead of liposomes did not lead to any detectable chiral supra-molecular assembly. However, it cannot be excluded if one allows the presence of the liposome interface to alter mβCD solubility and then promote the detachment and subsequent formation of a sparse population of extended microcrystalline structures. Such structures would also have to contain lipids, as both the mβCD response and the lipid (P–O stretch) response in SFS behave in the same manner, and they would have to be specific for POPS, not for POPC, which further reduces the likelihood of such a crystallization scenario. In the proposed supra-structure, the molecular dimensions of a POPS lipid and a mβCD molecular cavity (Figures 4 and S6D) are such that a complex of several stacked mβCDs can contain one or possibly two lipids as well a ring of water molecules. Cyclodextrins crystallize in cylindrical P2_1_ structures that are ∼ 0.8 nm thick.^74–76^ It is thus reasonable to assume that the interfacial self-assembly of mβCD shares similarities with the aggregation behavior of CD crystals in aqueous solutions. Such a mβCD-lipid–water complex further grows into a longer self-assembled structure, resulting in the peculiar SHS patterns of Figure 2D.

**Figure 4:**
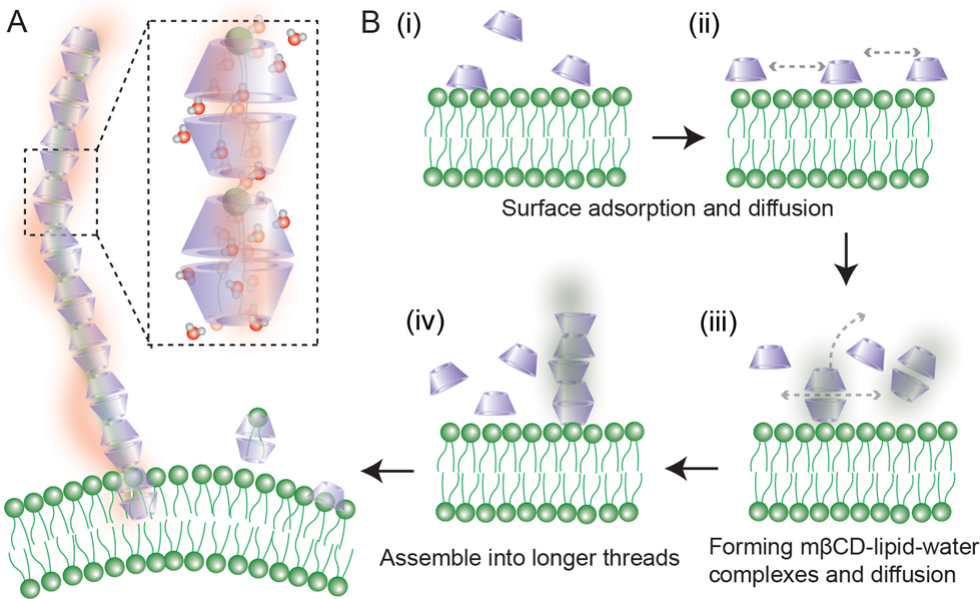
Suggested mechanism for the formation of extended chiral ordered water structures. A: Schematic illustration of an extended rod-like self-assembly complex formed by mβCD, lipids, and water molecules, where the red (green) shade shows the chiral arrangement of water (lipids). The dashed box shows a zoom-in of a potential self-assembly complex formed by mβCD, lipids, and water molecules. B: Illustration of a possible self-assembly scheme of mβCD on the lipid membrane surface. First, mβCD molecules are adsorbed onto the membrane interface (i), and the adsorbed mβCD molecules subsequently diffuse along the lipid membrane surface (ii). mβCD-lipid–water complexes can form at the interface, and they can diffuse along or leave the membrane surface (iii). With increased incubation time, complexes can merge leading to threads/wires of different sizes that protrude out of the membrane interface (iv).

To confirm the self-assembly hypothesis, atomic force microscopy (AFM) was used to image POPS lipid bilayers deposited on a mica substrate in the presence of mβCD (Figure 5), after 24-h incubation. The deposition is carried out from samples prepared identically as for SHS experiments, with the imaging conducted in solution (see SI, S1 for details). Although the bilayer is supported and effectively held in two dimensions, a thin water layer underneath the membrane^77^ allows for lateral diffusion within the membrane albeit at reduced rate (see ref.^78^ and refs therein). When imaging at low magnification (Figure 5A), many mβCD domains are visible with sizes ranging from 50 to 500 nm. The domains are mobile and can be disrupted by scanning the AFM tip. Photothermal off-resonance tapping^79^ was therefore preferred as an imaging mode to preserve the sample as much as possible. Profiles taken over selected domains (Figure 5B) show that their thickness is typically a multiple of 7.9 Å, that of a cyclodextrin (Figure 1A). Occasional small height variations are visible (single cyclodextrin), but these are easily removed by the tip, and therefore less stable. Control experiments conducted with only a POPS membrane (Figure 5C) or only mβCD deposited on the mica substrate (Figure 5D) do not show domains comparable to those visible in Figure 5A,B.

**Figure 5:**
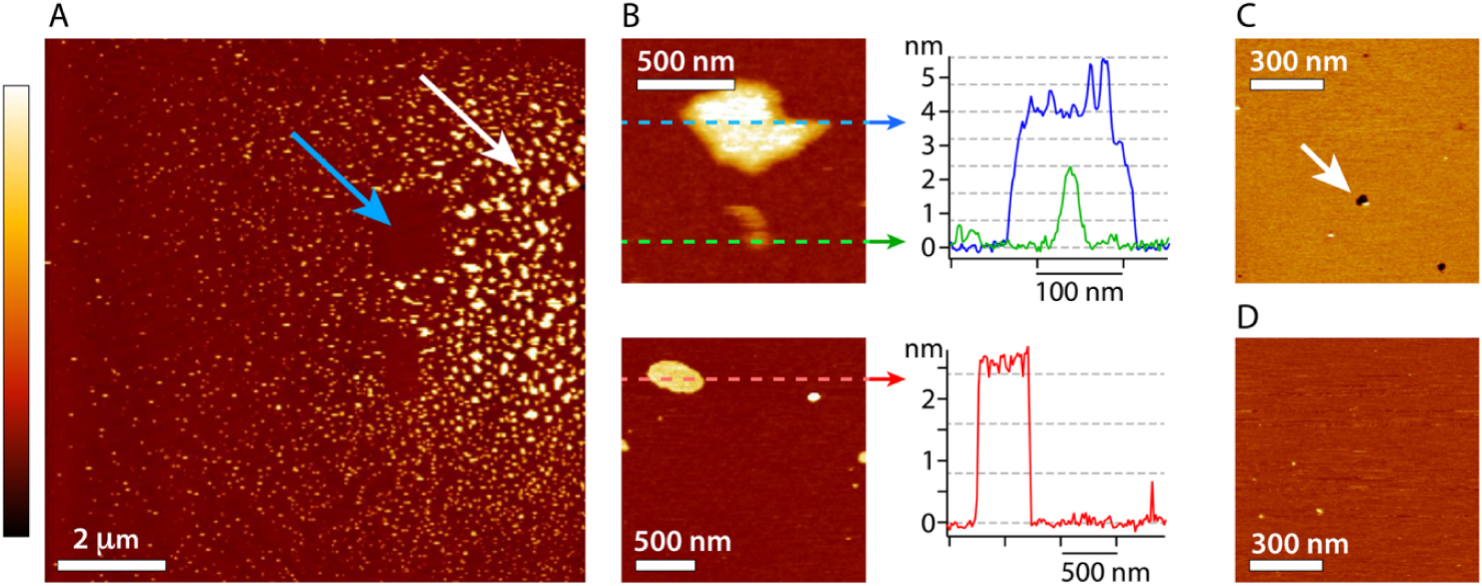
mβCD assemblies on POPS bilayers observed by AFM in aqueous solution. A: Typical low magnification topographical image of the membrane reveals many mβCD nanodomains (bright islands). Some regions of the membrane exhibit larger domains and in higher density (white arrow), but the domains remain mobile and can be easily swept aside by the AFM tip (blue arrow). B: Magnified view of selected regions with profiles taken over different nanodomains. The height of the mβCD structures suggest a preference for multiples of 7.9 Å (dashed gray horizontal lines), consistent with stacks of mβCDs. C: Control experiment on a POPS membrane without mβCD present. Occasional defects ∼ 5 nm deep (arrow) confirm a stable bilayer otherwise completely flat. D: Control experiment with mβCD directly onto the mica substrate, without lipids. No large or stable nanodomain are visible. The color scale bar represents a height variation of 6 nm (A, B, top, C, and D) and 4 nm (B, bottom).

Therefore, AFM images support the self-assembly mechanism of Figure 4 derived from experimental scattering (SHS, SFS, and DLS) data, which illustrates the formation of fragile/flexible self-assembled mβCD-lipid–water complexes that have heterogeneous size distributions on top of a lipid membrane. The micron long structures detected in the SHS experiments that grow out of the POPS liposomes surface are not expected to appear in AFM images, as the substrate-supported lipid bilayer, being essentially an infinite planar structure, likely offers different growth kinetic routes. In contrast, ∼ 56 nm radius liposomes have a limited membrane area, and the structure growth more easily takes place along the normal/radial direction, which is inhibited for supported lipid membranes.

Although there is insufficient information to understand all the details of the molecular mechanism, the picture obtained from the combined MD simulations, nonlinear light scattering, and AFM experiments clearly shows the formation of a long, thin structure with chiral water molecules that extend over several microns and are highly ordered, spectroscopically ice-like. Being extended over several microns, such chiral microenvironments may have been important steppingstones in the emergence of an enantioselective biochemical machinery.

## Conclusions

Using MD simulations, DLS, SHS, vibrational SFS, and AFM, the self-assembly of chiral cyclic poly sugars in solution and at nanoscale liposome interfaces in solution was investigated. MD simulations showed that a chiral water arrangement can form within a mβCD dimer. On the surface of POPS liposomes with mβCD, we observed from SHS patterns that interfacial water forms a thin long chiral suprastructure extending to over several microns in length. Vibrational SFS further revealed that water in these self-assembled complexes is highly ordered and spectroscopically ice-like. The SFS spectra also showed that mβCD forms chiral structures on the liposomes, but only when an out-of-plane H-bonding interaction can be made involving the lipids and the water.

Up until now, chiral interfacial water was known to exist within a monolayer around the molecular interfaces of DNA and peptides that were crafted/deposited onto a surface (see e.g., refs. 22,80 ). The present work shows that hydrating water molecules within the internal cavity of mβCD complexes can form extended chiral suprastructures up to 10 μm (along the direction of the main molecular axis), even leading to highly ordered spectroscopically ice-like water at room temperature. Water molecules in such structures exhibit slightly larger O–O distances than that in ice, making for a more open extended microenvironment which could have been important in the emergence of chiral specificity, as it would have formed a microenvironment for chiral amplification. Furthermore, the molecules that are needed to form this environment are relatively simple and would have been around before the emergence of more complex molecules such as DNA.^81^

## Supplementary Material





## Data Availability

The data that support the findings of this study are available from the corresponding author upon reasonable request.

## Notes

Sample preparation and characterization; MD simulation construction and analysis; SHS theory and experiment; SFS experiment; the procedure to obtain SFS water spectra; AFM method; computing SHS patterns; SFS study of the lipid headgroup and mβCD-lipid alkyl groups; global fitting of water spectra (PDF)
A spiral arrangement of water (MP4)
