# Supplementary material for: Interfacial Self-Assembly of Sugars at Nanoscale Membranes Leads to Micron-Scale, Spectroscopically Ice-Like Chiral Suprastructures of Water
Source: J Am Chem Soc. 2025 Sep 4;147(37):33413–23. doi: 10.1021/jacs.5c05215 (PMC12447511; doi:10.1021/jacs.5c05215)
Supplement: Supplementary file 1 [file ja5c05215_si_001.pdf]

Supporting Information

for

Interfacial self-assembly of sugars at nanoscale membranes leads to micron-scale, spectroscopically ice-like chiral suprastructures of water

Li Zhang[a], Jinchan Liu[b], Kislon Voïtchovsky[c], Chaudhary E. Rani[a], Saranya Pullanchery[a], Jan Dedic[a], Victor S. Batista[d], Georg E. Fantner[e], and Sylvie Roke[a,f,g,*]

[a]Laboratory for Fundamental BioPhotonics (LBP), Institute of Bioengineering (IBI), School of Engineering (STI), École Polytechnique Fédérale de Lausanne (EPFL), Lausanne, CH-1015, Switzerland; [b]Department of Molecular Biophysics and Biochemistry, Yale University, New Haven, CT06520, USA; [c]Physics Department, Durham University, Durham, DH1 3LE, UK; [d]Department of Chemistry, Yale University, CT06520, New Haven, USA; [e]Laboratory for Bio and Nano Instrumentation (LBNI), Institute of Bioengineering, School of Engineering, École Polytechnique Fédérale de Lausanne (EPFL), Lausanne, CH-1015, Switzerland; [f]Institute of Materials Science and Engineering (IMX), School of Engineering (STI), École Polytechnique Fédérale de Lausanne (EPFL), Lausanne, CH-1015, Switzerland; and [g]Lausanne Centre for Ultrafast Science, École Polytechnique Fédérale de Lausanne (EPFL), Lausanne, CH-1015, Switzerland.

[*]To whom correspondence may be addressed. Email: sylvie.roke@epfl.ch.

Supporting Information:

S1. Materials and Methods

S2. SHS Theory

S3. Computing SHS Patterns

S4. SFS Spectra and Global Fits

Supporting Figures:

Figure S1: Size distribution of POPS liposomes.

Figure S2: Measured IR and SF spectra and converted SFS water spectrum.

Figure S3. Parameters for Eq. S3.

Figure S4: Geometry of SHS from a cylindrical particle.

Figure S5: SHS patterns of POPC liposomes.

Figure S6: SFS spectra in the P-O and C-H stretch regions.

Figure S7: Global fits of the SFS water spectra in Figs. 3C and 3D.

Supporting Tables:

Table S1: Analytical expressions of the surface and particle susceptibility elements used for computing Eq. S4 and S5.

Table S2: Analytical expressions of the form factor functions and the scattering vector used for computing Eq. S4 and S5.

Table S3: Parameters used to obtain the solid curves in in Figs. 2C, S5A and S5C (black).

Table S4: Parameters used to obtain the solid curves in Fig. 2D, S5C (blue) and S5D (blue).

Table S5: Parameters used to fit the P-O spectra in Fig. S6A.

Table S6: Parameters used to fit the C-H spectra in Fig. S6B.

Table S7. Parameters used to fit the O-D spectra in Fig. S7.

S1. Materials and Methods

Chemicals. Methyl-β-cyclodextrin (mβCD, ≥98%) was purchased from Sigma Aldrich and used as received. The degree of methylation is 1.7-1.9 CH_3 per glucose unit. The molecular structure and shape of mβCD are shown in Fig. 1A (also Fig. S6D). The lipids, 1-palmitoyl-2-oleoyl-sn-glycero-3-phospho-L-serine (POPS) and 1-palmitoyl-2-oleoyl-glycero-3-phosphocholine (POPC), were purchased in powder form (>99%) from Avanti Polar Lipids (Alabama, USA) and stored at -20 °C. The chemical structures of the lipid headgroups are shown in Fig. 2A (inset, also Fig. S6D). Sodium Chloride (NaCl, 99.999 %) was purchased from ABCR GmbH, Germany; chloroform ($CHCl_3$) for analysis (Reag. Ph. Eur) was purchased from Merck, Germany; and heavy water (D_2O, 99.8% D) was purchased from Thermo Scientific; double heavy water, deuterium oxide-^{18}O (99% D, 95% ^{18}O) was purchased from Sigma Aldrich, which were used as received. Ultrapure H_2O with a resistivity of 18.2 MΩ·cm^{-1} was obtained from a Milli-Q UF plus instrument (Millipore Inc.).

Cleaning procedure. Glassware for liposome preparation was cleaned with a Deconex® (Borer Chemie AG) solution prepared by 1:20 dilution with ultrapure water (Milli-Q UF plus, Millipore, Inc., electrical resistance of 18.2 MΩ cm), and then rinsed thoroughly with ultrapure water at least 15 times.

Sample preparation. Liposomes were prepared by extrusion of rehydrated multilamellar vesicles according to the protocols described in Refs [1,2]. Briefly, a weighted amount of lipid powder was dissolved in $CHCl_3$ in a round-bottom glass container. A lipid film was formed on the glass surface by rotary evaporation of the solution under N_2 flow. The containers were placed under vacuum for at least 2 hours to evaporate the remaining $CHCl_3$. The film was then rehydrated in water (D_2O or $D_2{}^{18}O$) with 25 mM NaCl. The dispersion was sonicated and vortexed. Liposomes were formed by extruding the dispersion 30-50 times through a polycarbonate membrane with a pore size of 100 nm above the transition temperature of the lipid using a mini-extruder (Avanti Polar Lipids, Al, USA). The size distribution of the resulting liposomes was determined by dynamic light scattering (DLS) using a Malvern ZS nano-sizer instrument (Malvern ZS nano-sizer). The liposome stock solutions were stored in closed containers at 4 °C and used within a week after preparation. The Z-average of the diameter of the extruded POPS liposomes is ~115 nm with a polydispersity index (PDI) of < 0.1; an example of which is shown in Fig. S1 and Fig. 2B.

The liposome stock solutions were mixed with a mβCD solution in water or in an aqueous solution containing 25 mM NaCl. The final concentrations of lipid and mβCD was 5 mg/mL / 1 mM, respectively. The samples were stored in the dark and incubated in closed plastic containers at room temperature for ~24 hours or longer. The stability of the samples after incubation was verified by DLS before / after the SHS / SFS measurements. Each time 3 DLS measurements consisting of at least 10 runs were performed and their results were averaged.

For SHS and DLS measurements the samples were diluted 10× with 25 mM NaCl aqueous solutions prior to the measurement for a final lipid concentration of 0.5 mg/mL. For SFS measurements the samples were used undiluted.

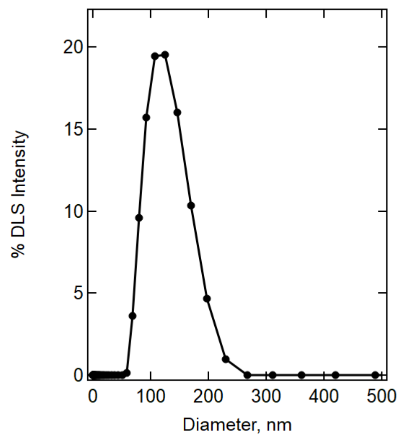

Figure S1: **Size distribution of POPS liposomes.** The DLS intensity diameter distribution of POPS liposomes.

Molecular dynamics (MD) system construction and conformation sampling. Our all-atom systems were built using a 1.25 Å resolution X-ray crystal structure (PDB ID: 6xx2), with a mβCD co-crystallized [3]. Using psfgen, we fully methylated the βCD by adding methyl groups to the O2 and O6 positions of each glucose unit and then minimized the modified monomer. To sample possible interactions between mβCD monomers, we assembled two mβCD monomers into three initial configurations: (1) Two secondary (larger) rims facing each other; (2) two primary (smaller) rims facing each other; (3) the primary rim of one mβCD facing the secondary rim of the other. For each configuration, we performed energy minimization for 1000 steps, followed by 1 ns of MD simulations. This process was repeated 10 times for each to search for stable conformation. Out of the 3 × 10 trials, we identified a stable conformation for the (1) configuration, where the secondary rims faced each other. This conformation was stabilized by 14 pairs of hydrogen bonds, with the methylated O2 groups acting as hydrogen bond acceptors and the O3 groups as donors (Fig. 1A). We further minimized the stable conformation for 50,000 steps. Afterward, we solvated the mβCD dimer with TIP3P water molecules [4] and 0.15 M NaCl. This solvated system of 5734 atoms with a 40 Å × 40 Å × 40 Å simulation box was then used to study the water structure inside the mβCD assembly.

MD protocol and water pattern analysis. The solvated structure was equilibrated for 1 ns and 10 replicas of 50-ns simulations were performed to study the water dynamics. We constrained the non-hydrogen atoms in mβCD, mimicking the assembly of mβCD, and analyzed the water structure in the mβCD assembly by calculating the water density. The water density was calculated by counting the occurrence of atoms within a unit box (0.106 Å × 0.106 Å × 0.106 Å, corresponding to 2 Bohr units) and normalizing by the total number of frames. All MD simulations were performed using NAMD [5] with the CHARMM36 force field for mβCD[6]. We maintained a constant temperature of 300 K using

Langevin dynamics (damping constant $\gamma = 1.0$ ps^{-1}) and enforced constant pressure (1 atm) using an anisotropic Langevin piston barostat [7]. We used a time step of 2 fs, calculating bonded and short-range nonbonded interactions (within a 12-Å cutoff) at each step. Long-range interactions were computed using the Particle-Mesh Ewald (PME) method, updated every other step [8]. All simulation results were analyzed using visual molecular dynamics (VMD) [9].

Second Harmonic Scattering (SHS). SHS measurements were performed using 190-fs laser pulses centered at 1028 nm with a 200 kHz repetition rate. The polarization of input pulses was controlled by a Glan-Taylor polarizer (GT10-B, Thorlabs) in combination with a zero-order half-wave plate (WPH05M-1030). The filtered (FEL0750, Thorlabs) input pulses with a pulse energy of 0.3 μJ (incident laser power P = 60 mW) were focused into a cylindrical glass sample cell (inner diameter 4.2 mm) with a waist diameter of ~35 μm and a Rayleigh length of 0.94 mm. The scattered second harmonic light was collected with a plano-convex lens (f = 5 cm), and then filtered (ZET514/10x, Chroma), polarized (GT10-A, Thorlabs), and finally focused into a gated photomultiplier tube (H7421-40, Hamamatsu). The angle of acceptance for the signal collection was 3.4°. The scattering pattern was measured at a scanning step of 5°, between -90° < θ < 90°. Each data point was acquired with an acquisition time of 20 × 1 s and a gate width of 10 ns. All measurements were performed in a temperature- and humidity-controlled room (T = 297 K; relative humidity, 26.0 %). The normalized SHS intensity $S(\theta)$ at the angle θ was calculated as:

$$S(\theta) = \frac{I(\theta)_{sample}^{XPP} - I(\theta)_{solvent}^{XPP}}{I(\theta)_{D2O}^{SSS}} \tag{S1}$$

where $I(\theta)_{sample}^{XPP}$ and $I(\theta)_{solvent}^{XPP}$ are the average SHS intensities of the sample and solvent at the same given temperature, respectively. $I(\theta)_{D_2O}^{SSS}$ is the average incoherent isotropic SHS intensity of water at room temperature. The X stands for the polarization state of the output beam relative to the scattering plane (P - parallel or S - perpendicular). Because bulk water is used as normalizing substance, all the measured patterns can be compared in their intensity with an error in the intensity of 1-2 %[10].

Vibrational Sum Frequency Scattering (SFS) Instrument. In the vibrational SFS spectroscopy system, IR and VIS pulsed laser beams are temporally and spatially overlapped inside the sample cuvette. The experimental set-up has been previously described in detail in Refs [11,12]. The broad IR pulses (3.4 μm, 10 μJ, with a full width at half maximum, FWHM, of 180 cm^{-1}) were obtained from an OPA system (HE-TOPAS-C, Light Conversion) with a Ti:sapphire femtosecond pump laser system (800 nm, 1 kHz, Spitfire Pro, Spectra physics). The output from the pump laser was split, with the first part being used to generate IR photons, as described, and the second part being directed into a home-built pulse shaper that generated the narrow VIS pulses (800 nm, 10 μJ, FWHM = 15 cm^{-1}). The

polarization of the IR beam was controlled by BaF_2 two wire-grid polarizers. The polarization of the VIS beam was controlled by a polarizer cube (CVI, PBS-800-050) and a half-wave plate (EKSMA, 460-4215). The IR and VIS beams were focused under an opening angle of 15° into a cuvette having CaF_2 (front) and quartz (back) windows. The cuvette has an optical path length of 200 μm. The emitted SF light was collected and collimated using a plano-convex lens (f = 15 mm, Thorlabs LA1540-B) at the scattering angle that aligned with the broad maximum intensity of the scattering patterns, which is at θ=57° as measured in air. The SFS light was spectrally filtered by two short-pass filters (3rd, Millenium, 3RD770SP) and its polarization was controlled with a Glan-Taylor prism (Thorlabs, GT15-B), after which it was spectrally dispersed with a monochromator (Acton, SpectraPro 2300i) and then detected by a gated intensified CCD camera (Princeton Instrument, PI-Max3) with a gate width of 10 ns. The acquisition time for a single spectrum was 600 s.

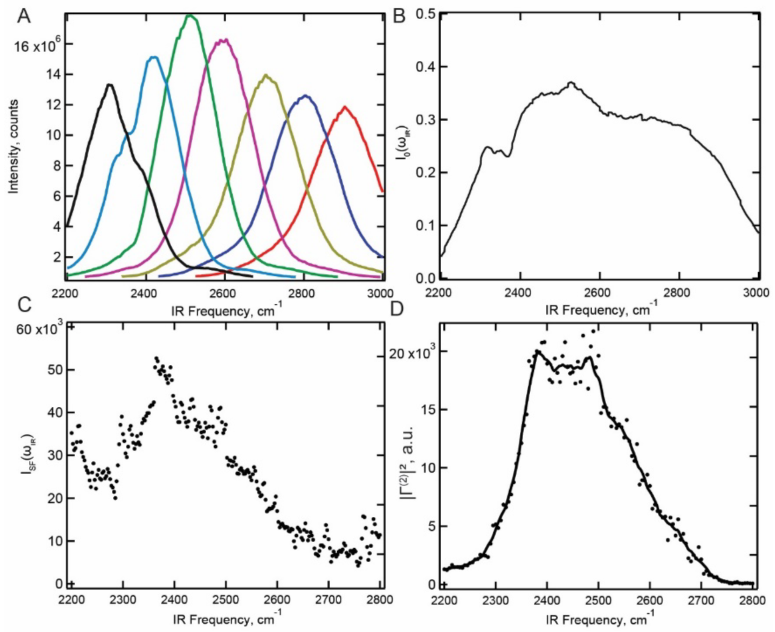

Figure S2: **Measured IR and SF spectra and converted SFS water spectrum**. Procedure for retrieving the interfacial water spectrum of POPS liposomes. A: The SFS signal from $BaTiO_3$ nanoparticle film, representing the shape of IR pulses used for SFS measurements, as well as B: the computed $I_0(\omega_{IR})$ spectrum. C: The total summed SFS spectrum (Eq. S2) for the entire O-D stretch region. D: The resultant $|\boldsymbol{\Gamma}^{(2)}|^2$ SF spectrum of POPS liposomes retrieved by dividing the SF measurement (C) by the function $C(\omega_{IR})$ (Eq. S3, Fig. S3C).

Vibrational SFS to record interfacial water spectra. Broadband IR pulses between 2200 and 2800 cm^{-1} spaced with 100 cm^{-1} frequency steps were used to probe the entire O-D stretching region. The infrared spectral profiles were recorded as the sum frequency intensity from a solid film of $BaTiO_3$ nanoparticles and are shown in Fig. S2A. The computed $I_0(\omega_{IR})$ is shown in Fig. S2B. The measured sum frequency spectrum at each frequency range was background subtracted and then normalized with IR and VIS pulse energy and acquisition time. The total SF intensity at each pulse center frequency ω_0 was computed as a weighted sum:

$$I_{SF(\omega=\omega_0)} = \frac{\sum_{i=1}^{n} I_{SF,i(\omega_0)}\, I_{0(\omega_{IR}),i}}{\sum_{i=1}^{n} I_{0(\omega_{IR}),i}} \quad \text{(S2)}$$

where the index i runs through all IR excitations that contribute to the intensity at $\omega = \omega_0$ and $I_{0(\omega_{IR}),i}$ is the incident IR intensity that generates sum frequency signal at $\omega = \omega_0$. The resultant spectrum after the summation is shown in Fig. S2C.

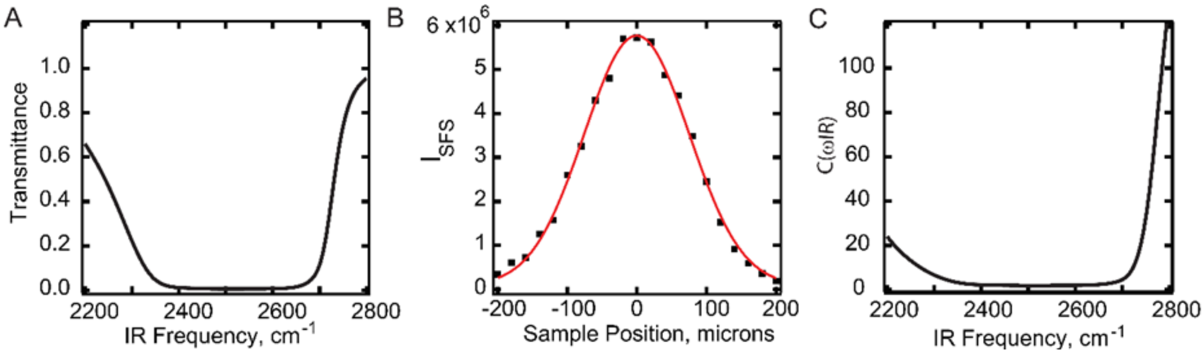

Figure S3: Parameters for Eq. S3. A: FTIR transmittance profile of D_2O measured using sample cell similar to the one used for SFS measurements but with a 10 µm path length. B: the integrated spectral intensities and the fit function that was used as $f_{focal}(z)$. C: $C(\omega_{IR})$ as computed from Eq. S3.

Converting the measured SF intensity to interfacial $|\boldsymbol{\Gamma}^{(2)}|^2$ spectra. The relation between SF intensity and the true nonlinear interfacial scattering response is given by the following equation:

$$\left|\boldsymbol{\Gamma}^{(2)}(\omega_{SF};\omega_{VIS},\omega_{IR})\right|^2 \propto \frac{I_{SF}(\omega_{SF};\omega_{VIS},\omega_{IR})}{\int_0^L I_{IR}(\omega_{IR},z) I_{VIS}(\omega_{VIS}) f_{focal}(z)\rho(z)\, dz} = \frac{I_{SF}(\omega_{IR})}{C(\omega_{IR})} \quad \text{(S3)}$$

where $\omega_{SF}, \omega_{VIS}$, and ω_{IR} are the frequencies of the sum frequency, visible, and infrared pulses. $I_{SF}(\omega_{SF};\omega_{VIS},\omega_{IR}) = I_{SF}(\omega_{IR})$ is the measured SF intensity. $I_{IR}(\omega_{IR}, z)$ is the intensity of the incident infrared light along the optical z axis which is given by Lambert-Beers' law, $I_{IR}(\omega_{IR}, z) = I_0(\omega_{IR})e^{-\alpha(\omega_{IR})z}$, with $I_0(\omega_{IR})$ being the incident IR intensity from the laser and $\alpha(\omega_{IR})$ the absorption spectrum. $\alpha(\omega_{IR})$ is computed from the measured IR transmission spectrum ($T(\omega_{IR})$, Fig. S3A) as $\alpha(\omega_{IR}) = \frac{-ln\,(T(\omega_{IR}))}{L}$. $I_{VIS}(\omega_{VIS})$ is the intensity of the incident visible light, $f_{focal}(z)$ is a measured collection function that describes the efficiency of light collection along the optical z axis of the collection optics (Fig. S3B), and $\rho(z)$ is the number density of particles at a specific depth, which is assumed to be uniform (i.e. $\rho(z) = 1$). $C(\omega_{IR})$ is the final correction factor resulting from numerically integrating the denominator in the middle of Eq. S3 (Fig. S3C). $\boldsymbol{\Gamma}^{(2)}(\omega_{SF};\omega_{VIS},\omega_{IR}) = \boldsymbol{\Gamma}^{(2)}$ is the effective second-order particle susceptibility that describes the spectral interfacial response

of droplets dispersed in solution. $\boldsymbol{\Gamma}^{(2)}$ is a function of the scattering angle (θ, defined as the angle between the scattered SF wavevector and that of the phase-matched direction, Fig. 3A), the size of the droplets (typically indicated by R, the average droplet radius), and the second-order surface susceptibility ($\boldsymbol{\chi}^{(2)}$) [13-15].

Eq. S3 contains a number of parameters that were experimentally determined. The absorption coefficient $\alpha(\omega_{IR})$, was computed from the transmission spectra of water. The transmission spectrum was recorded using a Bruker Vertex 70 FTIR spectrometer, with a transmission sample cell that has CaF_2 (front) and quartz (back) windows with an optical path length of 10 microns. The windows are the same windows as used for the SFS sample cell. The transmittance spectrum is shown in Fig. S3A. Another parameter is $f_{focal}(z)$, the collection function that describes the efficiency of light collection along the optical axis z of the collection optics. This function was measured using a sample of stearyl covered silica particles (R=123 nm) dispersed in CCl_4.[16] By recording the SFS spectrum while translating the sample along the optical axis (see also Ref. [17]), $f_{focal}(z)$, was determined. Fig. S3B shows the integrated spectral intensities and the fit function that was used as $f_{focal}(z)$. Fig. S3C shows the computed correction factor $C(\omega_{IR})$ from Eq. S3. The measured SF intensity and the corrected $\left|\boldsymbol{\Gamma}^{(2)}\right|^2$ spectra are shown in Fig. S2C and S2D respectively.

Atomic force microscopy (AFM) method. AFM experiments were conducted on a bespoke machine[18] capable of photothermal cantilever excitation, both on and off resonance. The more delicate measurements of the mβCD nanodomains were conducted with photothermal off-resonance tapping (PORT) using ultrasmall cantilevers with a nominal spring constant of 0.6 N/m (USC-F1.5-k0.6, Nanoworld, Neuchâtel, Switzerland). The PORT measurements ensured limited lateral disruption of the membrane and a good force control with minimal pressure on the sample[18]. All the measurements were conducted in a 25 mM NaCl solution, as for SHS measurements. The AFM measurements were repeated three times on different days to ensure reproducibility.

The sample preparation for AFM measurement was conducted by adsorption and rupture of SUVs, a procedure well-established[19]. In short, ~100 μL of a solution containing POPS SUVs (~10 mg/mL) previously incubated for at least 24 h with 1 mM mβCD was placed onto a disc of freshly cleaved mica and allowed to rest for 20 min at room temperature so as to form a supported lipid bilayer. The relatively high lipid concentration ensures uniform coverage of the substrate. More (25 mM NaCl) solution was subsequently added to the sample prior to imaging with the AFM.

S2. SHS Theory

SHS from spherical particles. The computed $S(\theta)$ curves can be obtained using nonlinear light scattering theory, described in detail in Refs. [20,21]. The equations used to relate $S(\theta)$ to the surface potential are derived using the Rayleigh-Gans-Debye (RGD) approximation [13], in the absence of

multiple scattering, with a first-order correction for the jump in the electromagnetic field amplitude across the interface[22].The scattered SH polarized light is a function of the geometry of the illuminating electromagnetic fields and their respective polarization state P or S), the shape (sphere) and radius (R) of the particles, and the effective second- and third-order nonlinear particle susceptibility ($\boldsymbol{\Gamma}^{(2)}$and $\boldsymbol{\Gamma}^{(3)}$), nonlinear nanodroplet properties. Within the RGD approximation and considering that we are dealing with an isotropic sphere, with an isotropic interface, in an isotropic outer medium, Gonella et al. [20] obtained an analytical solution for the ratio of the coherent SH response of the nanodroplets recorded in either the PPP or PSS polarization combination to the incoherent response of neat water recorded in the SSS polarization combination:

$$S_{PPP}(\theta) = \frac{I_{PPP}(\theta)}{I_{SSS}(\theta)} = \frac{\varepsilon_0^2\left(E_P(\omega)^2\left[cos\left(\frac{\theta}{2}\right)^3\left(\Gamma_1^{(2)}\right) + cos\left(\frac{\theta}{2}\right)\left(\Gamma_2^{(2)}+\Gamma_2^{(3)\prime}\right)(2cos(\theta)+1)\right]\right)^2}{\bar{\mu}^2 N_b/N_p} \quad \text{(S4)}$$

$$S_{PSS}(\theta) = \frac{I_{PSS}(\theta)}{I_{SSS}(\theta)} = \frac{\varepsilon_0^2\left(E_S(\omega)^2\left[cos\left(\frac{\theta}{2}\right)\left(\Gamma_2^{(2)}+\Gamma_2^{(3)\prime}\right)\right]\right)^2}{\bar{\mu}^2 N_b/N_p} \quad \text{(S5)}$$

where $\Gamma_i^{(2)}$and $\Gamma_2^{(3)\prime}$ are the only non-zero elements of the nonlinear effective particle susceptibilities. $\bar{\mu}$ is the average induced second-order dipole moment. N_p is the number of droplets and N_b is the density of bulk water (3.34 x 10^{28} molecules/m^3). Water, with a known[23] nonlinear optical response, is used here as a reference. The four independent elements of the effective particle susceptibility for spheres are related to the surface susceptibility according to the expressions in Table S1. $\chi_{s,1}^{(2)''}$and $\chi_2^{(3)''}$are second and third-order susceptibilities that are corrected for changes in the refractive index following Ref. [24]. $F_{1,2,3}$ are analytical goniometric scattering form factors that depend on R, q and in the case of F_3, also on κ. All these parameters are known. Thus, the quantities in Eq. S4-S5 can be calculated. The analytical expressions for the non-zero tensor elements of the surface susceptibility ($\chi_{s,1}^{(2)''}$and $\chi_{s,2}^{(2)''}$), the double layer ($\chi_2^{(3)''}$), the form factor functions ($F_{1,2,3}$) and the scattering vector ($\boldsymbol{q}$) are also given in Table S1 and S2. Eqs. S4 and S5 are derived assuming that the liquid interface is spatially isotropic in the azimuthal direction that the optical process is lossless, and that water possesses a broad orientational distribution. These assumptions were validated in previous studies[20-22]. Although we employ the RGD assumption for the linear interaction, the nonlinear interaction contains a correction to account for the change in field amplitude when the optical electromagnetic fields cross the particle/water interface. Dadap et al. showed that a linear correction term to the second order susceptibility is sufficient to correct for the change in the electromagnetic field when it crosses the interface[25]. This correction for the susceptibility value is used here and listed in Table S2.

Table S1: Analytical expressions of the surface and particle susceptibility elements used for computing Eq. S4 and S5.

**The effective particle susceptibility**	**Surface susceptibility**
$\Gamma_1^{(2)} = (2F_1(qR) - 5F_2(qR))\chi_{s,1}^{(2)''}$	$\chi_{s,1}^{(2)''} = 27\eta \frac{\left(\chi_{s,1}^{(2)}\eta^2 + 3\chi_{s,2}^{(2)}(\eta^2 - 1)\right)}{(2+\eta)^3}, \eta = \left(\frac{n_p}{n_{H_2O}}\right)^2$
$\Gamma_2^{(2)} = F_2(qR)\chi_{s,1}^{(2)''} + 2F_1(qR)\chi_{s,2}^{(2)''}$	$\chi_{s,2}^{(2)''} = 27\eta \frac{\chi_{s,2}^{(2)}}{(2+\eta)^3}$
$\Gamma_1^{(3)\prime} = 0$	$\chi_2^{(3)''} = 27\eta \frac{\chi_2^{(3)\prime}}{(2+\eta)^3}, \chi_2^{(3)\prime} = \frac{N_b}{\varepsilon_0}\left(\bar{\beta}^{(3)} + \frac{\bar{\beta}^{(2)}\mu_{dc}}{3k_B T}\right)$
$\Gamma_2^{(3)\prime} = 2\chi_2^{(3)''}\Phi_0(F_1(qR) + F_3(qR, \kappa R))$	

Table S2: Analytical expressions of the form factor functions and the scattering vector used for computing Eq. S4 and S5.

**Form factor functions and scattering vector**
$F_1(qR) = 2\pi R^2 i \left(\frac{sin(qR)}{(qR)^2} - \frac{cos(qR)}{qR}\right)$
$F_2(qR) = 4\pi R^2 i \left(3\frac{sin(qR)}{(qR)^4} - 3\frac{cos(qR)}{(qR)^3} - \frac{sin(qR)}{(qR)^2}\right)$
$F_3(qR, \kappa R) = 2\pi R^2 i \frac{qRcos(qR) + \kappa Rsin(qR)}{(qR)^2 + (\kappa R)^2}$
$\boldsymbol{q} = \boldsymbol{k}_0 - 2\boldsymbol{k}_1, q = \left\lvert\frac{4\pi\eta_{D_2O}}{\lambda_{SH}} sin\frac{\theta}{2}\right\rvert$

SHS from cylindrical particles. The theory of SH scattering from cylindrical particles was published by J.I. Dadap[26] and de Beer [27]. For simplicity, we assume that the cylinder is made of non-centrosymmetric material whose crystallographic coordinates are aligned with the coordinates of the object ($r' = (x', y', z')$ or (ρ', φ', z'), Fig. S4). The approach to derive the scattered SH light differs from that for spheres shown above. Following the approach by Dadap[26], the nonlinear polarization of a cylinder can be calculated as:

$$\mathbf{p}' = \frac{1}{V}\int_V \boldsymbol{\chi}^{(2)}(\boldsymbol{r}') \exp(i\boldsymbol{q} \cdot \boldsymbol{r}')\, d\boldsymbol{r}' \qquad \text{(S6)}$$

where V is the volume of the cylinder. The scattering wavevector and its magnitude are defined the same way as before: $\boldsymbol{q} = \boldsymbol{k}_0 - 2\boldsymbol{k}_1$ and $q = k_0 \sin(\theta/2)$. The geometry of the scattering process is shown in Fig. S4. The lab frame is represented by a Cartesian coordinate system $(\hat{\boldsymbol{x}}, \hat{\boldsymbol{y}}, \hat{\boldsymbol{z}})$ while the particle is represented by a rotated coordinate system $(\hat{\boldsymbol{x}}', \hat{\boldsymbol{y}}', \hat{\boldsymbol{z}}')$. The cylinder can be arbitrarily rotated relative to the lab frame via the Euler matrix:

$$\boldsymbol{M}(\alpha, \beta, 0) = \boldsymbol{R}_x(\beta)\boldsymbol{R}_z(\alpha) \qquad \text{(S7)}$$

where $\boldsymbol{R}_i(j)$ is a matrix representing the rotation by an angle j around an axis i. We can use the axial symmetry axis of the cylinder to define a cylindrical coordinate system with unit vectors $(\hat{\boldsymbol{\rho}}', \hat{\boldsymbol{\varphi}}', \hat{\boldsymbol{z}}')$ which are related to the cylinder's Cartesian coordinates $(\hat{\boldsymbol{x}}', \hat{\boldsymbol{y}}', \hat{\boldsymbol{z}}')$ via the transformations:

$$\hat{\boldsymbol{\rho}}' = \cos(\varphi')\,\hat{\boldsymbol{x}}' + \sin(\varphi')\,\hat{\boldsymbol{y}}'$$
$$\hat{\boldsymbol{\varphi}}' = -\sin(\varphi')\,\hat{\boldsymbol{x}}' + cos(\varphi')\hat{\boldsymbol{y}}' \qquad \text{(S8)}$$
$$\hat{\boldsymbol{z}}' = \hat{\boldsymbol{z}}'$$

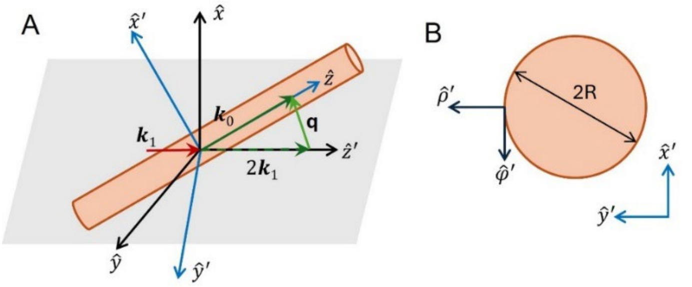

Figure S4: Geometry of SHS from a cylindrical particle. A: A perspective view of the scattering geometry. The laboratory Cartesian coordinates $(\hat{\boldsymbol{x}}, \hat{\boldsymbol{y}}, \hat{\boldsymbol{z}})$ are shown in black, the particle's Cartesian coordinate $(\hat{\boldsymbol{x}}', \hat{\boldsymbol{y}}', \hat{\boldsymbol{z}}')$ are shown in blue and are related to the laboratory coordinates via an arbitrary rotation using the Euler angles α, β. The scattering plane is defined by the vectors $\hat{\boldsymbol{x}}$ and $\hat{\boldsymbol{z}}$. The wavevector of the incident light $\boldsymbol{k}_1$ is in the $\hat{\boldsymbol{z}}$ direction. B: Top view of the cylinder. The orthogonal unit vectors $\hat{\boldsymbol{\rho}}'$ lies in the radial direction, the unit vector $\hat{\boldsymbol{\varphi}}'$ is normal to the surface and is orthogonal to $\hat{\boldsymbol{\rho}}'$ and $\hat{\boldsymbol{z}}'$.

The source vector $\boldsymbol{r}'$ can be expressed in the Cartesian or cylindrical coordinates of the particle as:

$$\boldsymbol{r}' = x'\hat{\boldsymbol{x}}' + y'\hat{\boldsymbol{y}}' + z'\hat{\boldsymbol{z}}' = \rho'\hat{\boldsymbol{\rho}}' + z'\hat{\boldsymbol{z}}' \qquad \text{(S9)}$$

The electric field in the lab and the particle frames is:

$$\hat{\boldsymbol{E}}_{\boldsymbol{0}} = E_{01}\hat{\boldsymbol{x}} + E_{02}\hat{\boldsymbol{y}} = E_1\hat{\boldsymbol{x}}' + E_2\hat{\boldsymbol{y}}' + E_3\hat{\boldsymbol{z}}' = E_\rho\hat{\boldsymbol{\rho}}' + E_\varphi\hat{\boldsymbol{\varphi}}' + E_3\hat{\boldsymbol{z}}' \qquad \text{(S10)}$$

The **E**-field components in the lab frame $(E_{01}, E_{02}, 0)$ and those in the particle frame (E_1, E_2, E_3) are related by a Euler rotation: $E_i = M_{ij}E_{0j}$. According to Eq. S8 the relationship between the **E**-field components in the particle's Cartesian and cylindrical coordinates is:

$$E_\rho = \cos(\varphi')\,E_1 + \sin(\varphi')\,E_2$$
$$E_\varphi = -\sin(\varphi')\,E_1 + cos(\varphi')E_2 \qquad \text{(S11)}$$

The wavevector can be expressed in the lab and the particle frame as:

$$\mathbf{q} = q_1\hat{\boldsymbol{x}} + q_2\hat{\boldsymbol{y}} + q_3\hat{\boldsymbol{z}} = Q_1\hat{\boldsymbol{x}}' + Q_2\hat{\boldsymbol{y}}' + Q_3\hat{\boldsymbol{z}}' \tag{S12}$$

where $(q_1, q_2, q_3) = -k_0(\sin(\theta), 0, \cos(\theta) - 1)$ and $Q_i = M'_{ij}q_j$.

For a cylinder made of a noncentrosymmetric medium, the SH signal is dominated by the bulk response $\boldsymbol{\chi_0}^{(2)} = \boldsymbol{\chi}^{(2)}: \hat{\boldsymbol{E}}_0\hat{\boldsymbol{E}}_0$ which is independent of $\boldsymbol{r}'$ and can be taken out of the integral in Eq. S6. The remaining terms in the integral give us the form factor *F*. To evaluate it, we need to express the term $\boldsymbol{q} \cdot \boldsymbol{r}'$ using Eqs. S9 and S12:

$$\boldsymbol{q} \cdot \boldsymbol{r}' = Q_1\rho' \cos(\varphi') + Q_2\rho' \sin(\varphi') + Q_3 z' \tag{S13}$$

The form factor *F* is then:

$$F = \frac{1}{V}\int_{-L/2}^{L/2}\int_0^{2\pi}\int_0^R \exp\left[i\left(Q_1\rho' \cos(\varphi') + Q_2\rho' \sin(\varphi') + Q_3 z'\right)\right]\rho' d\rho' d\varphi' dz' \\ = 2\frac{J_1(QR)}{QR}\frac{\sin(LQ_3/2)}{LQ_3/2} \tag{S14}$$

where J_1 is the Bessel function of the first kind and first order, $Q = \sqrt{Q_1^2 + Q_2^2}$, R is the radius of the cylinder, and L is its height. The polarization of the cylinder in its reference frame is:

$$\mathbf{p}' = F\boldsymbol{\chi_0}^{(2)} \tag{S15}$$

To calculate the detected E-field, we need to express $\mathbf{p}'$ in the lab frame as $\mathbf{p}$ via an inverse rotation: $\mathbf{p} = \boldsymbol{M}^T\mathbf{p}'$. The amplitude of the E-field at the detector is then proportional to:

$$E(2\omega) \propto \mathbf{p} \cdot \hat{\mathbf{u}} \tag{S16}$$

where $\hat{\mathbf{u}}$ is a unit polarization vector of the scattered light.

S3. Computing SHS Patterns

Spherical particles. The computed $S(\theta)$ curve for the SHS data recorded from POPS liposomes in the PPP polarization combination (Fig. 2C, black, achiral pattern of spherical shell) was obtained using Eqs. S1, S4, and S5 with the parameters in Table S3. The DLS measured POPS liposome diameter is ~113 ± 1 nm, so its radius is round to 56 nm for calculation. The same experiment in Fig. 2 was repeated for POPC liposomes. The resultant chiral and achiral SHS patterns for POPC (black) liposomes before and after the mβCD incubation are plotted in Fig. S5, together with POPS (blue) liposomes data for

comparison. Before incubation, the achiral response (Fig. S5A) shows characteristic two lobes SHS patterns for both POPC and POPS liposomes, but with a relatively weaker intensity for POPC due to its net charge neutrality. Meanwhile, within the detection limit, the chiral response is absent in an aqueous solution of both POPC and POPS liposomes. Both achiral SHS patterns are well described by the theory of nonlinear light scattering from spherical shells (see SI, S2). After adding mβCD to the POPC/POPS liposome solutions, the SHS patterns display drastic changes for POPS but not for POPC (Fig. S5C and S5D). Both achiral and chiral responses from POPC liposomes are not affected by mβCD. The DLS size distributions of POPC liposomes before and after 24-hours incubation with 1 mM mβCD are given in Fig. S5 inset, which shows no size change upon adding mβCD.

Table S3: Parameters used to obtain the solid curves in Figs. 2C, S5A and S5C (black).

**Parameter**
$\mu_{dc} = 8.97 \times 10^{-30}\ Cm$
SH wavelength, λ_{SH}: 514 nm
Refractive index of heavy water, n_{D_2O}: 1.33
Refractive index of liposomes, $n_{liposome}$: 1.4
Radius of liposomes, R: 56 nm (POPS),<br>63 nm (POPC)
Number density of liposomes, N_p: $1.1 \cdot 10^{12}$ mL^{-1}
Ionic strength of water: 25 mM

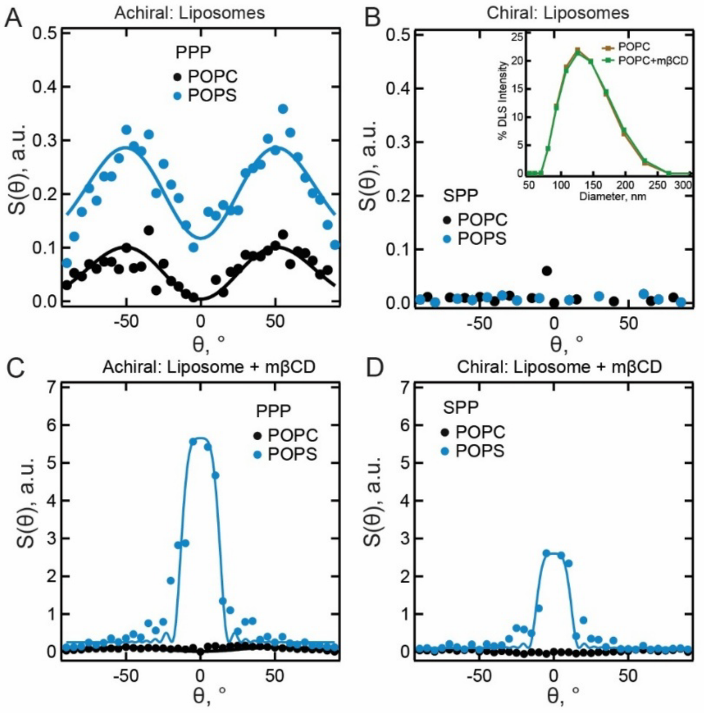

Figure S5: Comparing SHS patterns of POPC and POPS liposomes before and after the addition of mβCD. A, B: Achiral (A, PPP) and chiral (B, SPP) SHS patterns of POPC (black) and POPS (blue) liposomes in 25 mM NaCl aqueous solution. The solid lines are the scattering patterns computed using nonlinear light scattering theory for spherical particles (SI, S2, parameters in Table S3). The inset shows DLS size distributions of POPC liposomes before (brown, 0 h) and after (green, 24 h) 24-hour incubation with 1 mM mβCD. C, D: Achiral (C, PPP) and chiral (D, SPP) SHS patterns of POPC (black) and POPS

(blue) liposomes in 25 mM NaCl aqueous solution after 24-hour incubation with 1 mM mβCD. The solid lines are the scattering patterns computed using nonlinear light scattering theory for cylinders (SI, S3, parameters in Table S4).

Cylindrical particles. The solid lines in Fig. 2D and Figs. S5C and S5D display the computed SHS patterns from chiral and achiral cyclodextrin incubated POPS liposomes. Here, the theory of SH scattering from cylindrical particles was used (Eq. S6-S16). Since the values of the susceptibility ($\boldsymbol{\chi}^{(2)}$ or $\boldsymbol{\chi}_0{}^{(2)}$) tensor elements are unknown and there are too many unknowns to estimate them, the detected SHS intensity was calculated using

$$\mathrm{I}_{SHS}(2\omega, \theta) \propto |F|^2 \tag{S17}$$

where F is given as Eq. S14. The related parameters are given in Table S4. The radius of cylinder is set as 1 nm, which is on the order of magnitude of the width of an mβCD molecule (Fig. 1A). The fitted lengths from PPP and SPP patterns are 10025 ± 683 nm and 12672 ± 1020 nm, respectively, which are different but relatively close considering that this calculation provides only an approximate estimation. These lengths are insensitive to the radius value.

Table S4: Parameters used to obtain the solid curves in Fig. 2D, S5C (blue) and S5D (blue).

**Parameter**
$k_0 = 2\pi/\lambda_{SH}$
SH wavelength, λ_{SH}: 514 nm
$\alpha = 0$
$\beta = \pi/8$
Radius of cylinder, R: 1 nm
Length of cylinder, L: 10025 ± 683 nm for PPP
L: 12672 ± 1020 nm for SPP
Ionic strength of water: 25 mM

S4. SFS Spectra and Global Fits

SFS Study of the Lipid Headgroup and mβCD-Lipid Alkyl Groups

SFS spectra recorded in the P-O and C-H stretch regions report on the lipids in the liposomes (as mβCD does not contain P-O groups) and on both lipids and mβCD as both contain C-H groups. Fig. S6 shows SFS spectra for POPS liposomes in a 25 mM NaCl solution before (black traces) and after 24 hours of mβCD incubation (blue traces). POPC liposome spectra recorded after incubation are also shown for comparison (grey traces). Fig. S6A shows the P-O region and S6B shows the C-H spectral region. Fig. S6C shows the integrated SFS intensity in the C-H stretching region as a function of time. The SFS spectra were fitted using Levenberg-Marquadt iterations with Igor Pro 8 using the equation:

$$I_{SFS}(\omega_{IR}, \theta) \propto \left| A_{NR} e^{i\varphi_{NR}} + \sum_v \frac{A_v(\theta)\gamma_v}{\omega_{IR} - \omega_v + i\gamma_v} \right|^2 \tag{S18}$$

where A_{NR} and φ_{NR} are the amplitude, shape and phase of non-resonant background, $A_v(\theta)$, ω_v, and γ_v denote the amplitude, frequency and linewidth of resonant vibrational modes. The fit parameters for Fig. S6A and S6B are given as in Tables S5 and S6, respectively.

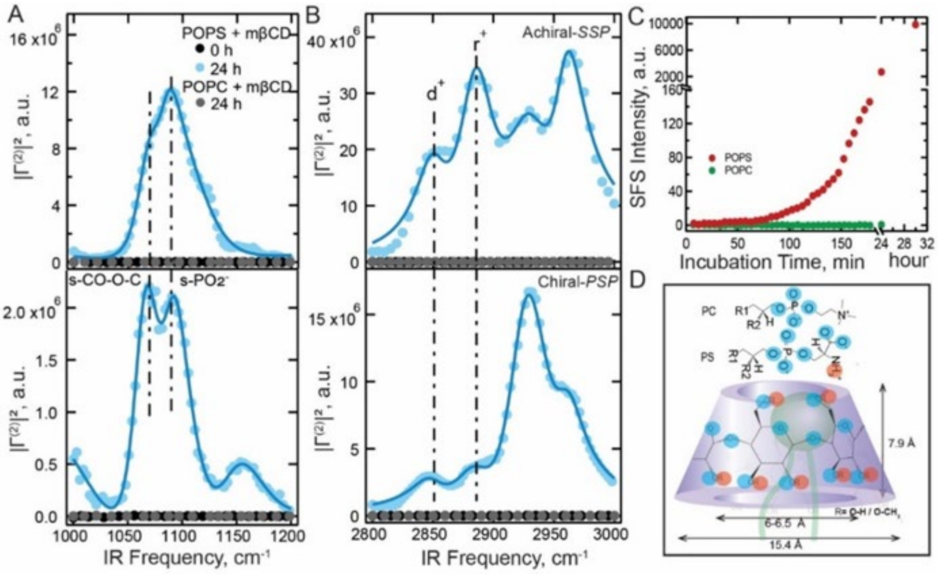

Figure S6: The SFS spectra in the P-O and C-H stretch region. A: The headgroup vibrational stretch spectra of POPS liposomes before (black, 0 h) and after 24-hour incubation (blue) with 1 mM mβCD, and the POPC liposomes spectra after 24-hour incubation (grey), measured using achiral (SSP, top) and chiral (PSP, bottom) polarization combinations. Solid lines are spectral fits obtained using the Eq. S18 in SI(S4). B: The C-H stretch spectra of POPS liposomes before (black) and after 24-hour incubation (blue), and the POPC lipsomes spectra after 24-hour incubation (grey) with 1 mM mβCD, measured using achiral (SSP, top) and chiral (PSP, bottom) polarization combinations. Solid lines are spectral fits obtained using the Eq. S18 in SI, S4. C: Integrated SFS intensity (measured using the SSP polarization combination) in the C-H stretch region (2800-3000 cm^{-1}) of POPS (red) and POPC (green) liposomes incubated with 1 mM mβCD as a function of incubation time. D: The structures of mβCD, PS, and PC lipid headgroups and a possible interaction scheme of PS lipids with mβCD. The possible H-bond donors (acceptors) on the lipid headgroups and mβCD are indicated in red (blue) circles. Note that groups marked as R can be an H or CH_3 on mβCD. Therefore, these are marked as potential H-bond donors.

Figs. S6A and S6B show the achiral (top) and chiral (bottom) vibrational SFS spectra for POPS and POPC liposomes in aqueous 25 mM NaCl solution before and after 24 hours of mβCD incubation, probing the vibrational modes of the headgroup region (P-O and O-C-O stretch modes, S6A) and the C-H stretch modes (S6B), respectively. Before incubation, no detectable spectral signatures were visible, as the alkyl chains and the headgroups were symmetrically distributed over the inner and outer leaflets, in agreement with previous work in Ref. [28]. After 24 hours of incubation the POPC spectra remained unchanged (grey traces). The POPS spectra have changed drastically, indicating that mβCD induces surface structural transformations on POPS liposomes but not on POPC liposomes in solution, consistent with SHS results (Figs. 2 and S5).

The symmetric stretch mode of the phosphate group ($\nu_s(PO_2^-)$), as well as the O-CO stretch mode [29-32], have both grown in intensity in both the chiral and the achiral responses for POPS liposomes

(Fig. S6A). The lipid head groups have thus adopted chiral arrangements, indicating that they interact with the cyclodextrins, e.g. by means of H-bonding. To illustrate how this might work, Fig. S6D shows the structure and dimensions of the mβCD cavity and two lipid headgroups, phosphatidylserine (PS) and phosphatidylcholine (PC). Based on the molecular structures of these three molecules, chiral complexation can occur between mβCD and PS but not with PC. PS can H-bond on 3 locations with mβCD, whereby H-bonds can be formed between the P-O group, the COO^- group, and the NH_3^+ group, with the -OH and C-O-C groups of the sugar. The possible H-bond donors and acceptors are marked as red and blue circles, respectively, also in Fig. 2A. The PC headgroup has only 2 possible H-bonding sites which excludes the formation of a chiral supra structure through interaction with mβCD.

Figure S6B shows a similar trend as Fig. S6A: after incubation, a variety of modes appear in the C-H stretch region of POPS liposomes that can be attributed to both the lipid and mβCD. The symmetric CH_2 (d^+) and CH_3 (r^+) are highlighted in the graphs. Further details on spectral assignments are given in Table S6. As both headgroup and alkyl group modes appear in the chiral SFS spectrum, it thus suggests that mβCD has transferred its chirality onto the liposome interface by interfacial intermolecular interactions. The SFS spectra in Figs. S6A and 6B and the integrated SFS responses in Fig. S6C reveals that POPC has no detectable interactions with mβCD, while POPS has. The steady growth of the intensity over time suggests that the interactions are slow, which can be attributed to a surface reorganization process, which requires the assembly of many molecules in a coordinated fashion. This growing trend determines that enough incubation (interaction) time (at least few hours) is needed to acquire significant chiral responses.

Table S5. Parameters used to fit the P-O spectra in Fig. S6A.

	SSP			PSP		
Mode	ω_v, cm^{-1}	γ_v, cm^{-1}	A_v, a.u.	ω_v, cm^{-1}	γ_v, cm^{-1}	A_v, a.u.
vs(CO-O-C)	1066.5	16.5	2324.6	1065	16.34	1535.7
vs(PO_2^-)	1085	13.55	1488.7	1090	17.44	1155.5
				1112	125.04	-2232
vs(PO_2^-), mβCD	1122	40	-1489.2			
mβCD				1146	33.73	1084.4
(A_{NR}, φ_{NR})	(896.47, -21°)			(1375.94, -73.13°)		

Table S6. Parameters used to fit the C-H spectra in Fig. S6B.

	SSP			PSP		
Mode	ω_v, cm^{-1}	γ_v, cm^{-1}	A_v, a.u.	ω_v, cm^{-1}	γ_v, cm^{-1}	A_v, a.u.
d^+	2851	16	2007.4	2850	16	665.44
r^+	2886	18	3874.7	2886	18	778.42
d^{+FR}	2909	20	299.8	2909	20	-589.1

d^-	2927	23	2714.9	2927	20	3262.5
r^{+FR}	2935	15	-26.9	2935	16	532.2
r^-	2959	15	3035.6	2960	15	1096.3
(A_{NR}, φ_{NR})	(1054.93, -45.12°)			(32.93, -65.61°)		

Global fits of water spectra.

Figure S7 shows the global fits as straight lines of the water spectra in Figs. 3C and 3D using Eq. (S18). The details of fitting parameters are given in Table S7.

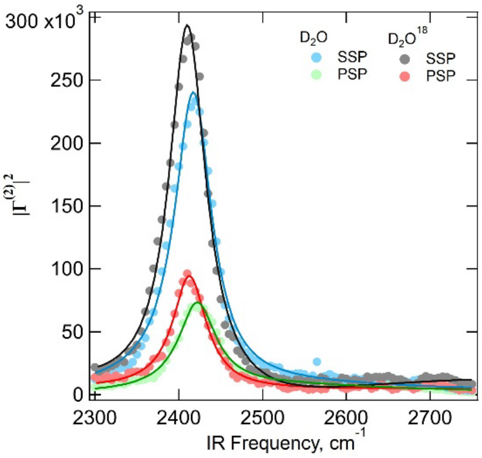

Figure S7: Global fits of the SFS water spectra in Figs. 3C and 3D. The solid lines are global fitting of the O-D spectra in Figs. 3C and 3D from POPS liposome with mβCD in D_2O at SSP (blue) and PSP (green) polarization combination, and in D_2O^{18} at SSP (black) and PSP (red) polarization combination, respectively.

Table S7. Parameters used to fit the O-D spectra in Fig. S7.

	SSP		PSP	
Parameter	D_2O	D_2O^{18}	D_2O	D_2O^{18}
ω_v, cm^{-1}	2418.1 ± 0	2410.1 ± 0	2422.4 ± 0.26	2412.3 ± 0.21
γ_v, cm^{-1}	27 ± 0	26.85 ± 0.21	26.909 ± 0.21	24.775 ± 0.20
A_v, a.u.	477.7 ± 7.63	485.79 ± 2.45	216.1 ± 3.07	245.54 ± 1.11
ω_v, cm^{-1}	2490 ± 0	2480 ± 0	2490 ± 0	2480 ± 0
γ_v, cm^{-1}	165 ± 0	165 ± 0	164.47 ± 6.07	165 ± 0
A_v, a.u.	64.98 ± 2.47	-85.56 ± 4.63	57.15 ± 5.36	-9.68 ± 2.58

References:

(1) Hope, M.; Bally, M.; Webb, G.; Cullis, P. Production of large unilamellar vesicles by a rapid extrusion procedure. Characterization of size distribution, trapped volume and ability to maintain a membrane potential. *Biochimica et Biophysica Acta (BBA)-Biomembranes* **1985**, *812*, 55-65.

(2) MacDonald, R. C.; MacDonald, R. I.; Menco, B. P. M.; Takeshita, K.; Subbarao, N. K.; Hu, L.-r. Small-volume extrusion apparatus for preparation of large, unilamellar vesicles. *Biochimica et Biophysica Acta (BBA)-Biomembranes* **1991**, *1061*, 297-303.

(3) Plaza-Garrido, M.; Salinas-García, M. C.; Martínez, J. C.; Cámara-Artigas, A. The effect of an engineered ATCUN motif on the structure and biophysical properties of the SH3 domain of c-Src tyrosine kinase. *J. Biol. Inorg. Chem.* **2020**, *25*, 621-634.

(4) Jorgensen, W. L.; Chandrasekhar, J.; Madura, J. D.; Impey, R. W.; Klein, M. L. Comparison of simple potential functions for simulating liquid water. *J. Chem. Phys.* **1983**, *79*, 926-935.

(5) Phillips, J. C.; Braun, R.; Wang, W.; Gumbart, J.; Tajkhorshid, E.; Villa, E.; Chipot, C.; Skeel, R. D.; Kalé, L.; Schulten, K. Scalable molecular dynamics with NAMD. *J. Comput. Chem.* **2005**, *26*, 1781-1802.

(6) Guvench, O.; Greene, S. N.; Kamath, G.; Brady, J. W.; Venable, R. M.; Pastor, R. W.; Mackerell, A. D. Additive empirical force field for hexopyranose monosaccharides. *J. Comput. Chem.* **2008**, *29*, 2543-2564.

(7) Martyna, G. J.; Tobias, D. J.; Klein, M. L. Constant pressure molecular dynamics algorithms. *J. Comput. Chem.***1994**, *101*, 4177-4189.

(8) Darden, T.; York, D.; Pedersen, L. Particle mesh Ewald: An N·log(N) method for Ewald sums in large systems. *J. Chem. Phys.* **1993**, *98*, 10089-10092.

(9) Humphrey, W.; Dalke, A.; Schulten, K. VMD: Visual molecular dynamics. *J. Mol. Graph. Model* **1996**, *14*, 33-38.

(10) Chen, Y.; Okur, H. I.; Gomopoulos, N.; Macias-Romero, C.; Cremer, P. S.; Petersen, P. B.; Tocci, G.; Wilkins, D. M.; Liang, C.; Ceriotti, M. Electrolytes induce long-range orientational order and free energy changes in the H-bond network of bulk water. *Sci. Adv.* **2016**, *2*, e1501891.

(11) de Aguiar, H. B.; Samson, J.-S.; Roke, S. Probing nanoscopic droplet interfaces in aqueous solution with vibrational sum-frequency scattering: A study of the effects of path length, droplet density and pulse energy. *Chem. Phys. Lett.* **2011**, *512*, 76-80.

(12) de Aguiar, H. B.; Scheu, R.; Jena, K. C.; de Beer, A. G. F.; Roke, S. Comparison of scattering and reflection SFG: a question of phase-matching. *Phys. Chem. Chem. Phys.* **2012**, *14*, 6826-6832.

(13) de Beer, A. G. F.; Roke, S. Sum frequency generation scattering from the interface of an isotropic particle: Geometrical and chiral effects. *Phys. Rev. B* **2007**, *75*, 245438.

(14) Roke, S.; Bonn, M.; Petukhov, A. V. Nonlinear optical scattering: The concept of effective susceptibility. *Phys. Rev. B* **2004**, *70*, 115106.

(15) Dadap, J. I.; de Aguiar, H. B.; Roke, S. Nonlinear light scattering from clusters and single particles. *J. Chem. Phys.* **2009**, *130*, 214710.

(16) Roke, S.; Berg, O.; Buitenhuis, J.; van Blaaderen, A.; Bonn, M. Surface molecular view of colloidal gelation. *Proc. Natl. Acad. Sci. U. S. A.* **2006**, *103*, 13310.

(17) Roke, S.; Roeterdink, W. G.; Wijnhoven, J. E. G. J.; Petukhov, A. V.; Kleyn, A. W.; Bonn, M. Vibrational sum frequency scattering from a submicron suspension. *Phys. Rev. Lett.* **2003**, *91*, 258302.

(18) Nievergelt, A. P., Banterle, N., Andany, S. H., Gönczy, P. & Fantner, G. E. High-speed photothermal off-resonance atomic force microscopy reveals assembly routes of centriolar scaffold protein SAS-6. *Nat Nanotech*. **2018**, *13*, 696-701.

(19) Reviakine, I. & Brisson, A. Formation of supported phospholipid bilayers from unilamellar vesicles investigated by atomic force microscopy. *Langmuir* **2000**, *16*, 1806-1815.

(20) Gonella, G.; Lütgebaucks, C.; de Beer, A. G. F.; Roke, S. Second harmonic and sum-frequency generation from aqueous interfaces is modulated by interference. *J. Phys. Chem. C* **2016**, *120*, 9165-9173.

(21) Lütgebaucks, C.; Gonella, G.; Roke, S. Optical label-free and model-free probe of the surface potential of nanoscale and microscopic objects in aqueous solution. *Phys. Rev. B* **2016**, *94*, 195410.

(22) de Beer, A. G. F.; Roke, S. Obtaining molecular orientation from second harmonic and sum frequency scattering experiments in water: Angular distribution and polarization dependence. *J. Chem. Phys.* **2010**, *132*, 234702.

(23) Gubskaya, A. V.; Kusalik, P. G. The multipole polarizabilities and hyperpolarizabilities of the water molecule in liquid state: an ab initio study. *Mol. Phys.* **2001**, *99*, 1107-1120.

(24) Dadap, J. I.; Shan, J.; Eisenthal, K. B.; Heinz, T. F. Second-harmonic Rayleigh scattering from a sphere of centrosymmetric material. *Phys. Rev. Lett.* **1999**, *83*, 4045.

(25) Dadap, J. I.; Shan, J.; Heinz, T. F. Theory of optical second-harmonic generation from a sphere of centrosymmetric material: small-particle limit. *J. Opt. Soc. Am. B* **2004**, *21*, 1328-1347.

(26) Dadap, J. I. Optical second-harmonic scattering from cylindrical particles. *Phys. Rev. B* **2008**, *78*, 205322.

(27) de Beer, A. G.; Roke, S.; Dadap, J. I. Theory of optical second-harmonic and sum-frequency scattering from arbitrarily shaped particles. *JOSA B* **2011**, *28*, 1374-1384.

(28) Smolentsev, N.; Lütgebaucks, C.; Okur, H. I.; De Beer, A. G. F.; Roke, S. Intermolecular headgroup interaction and hydration as driving forces for lipid transmembrane asymmetry. *J. Am. Chem. Soc.* **2016**, *138*, 4053-4060.

(29) Chen, X.; Hua, W.; Huang, Z.; Allen, H. C. Interfacial water structure associated with phospholipid membranes studied by phase-sensitive vibrational sum frequency generation spectroscopy. *J. Am. Chem. Soc.* **2010**, *132*, 11336-11342.

(30) Chen, Y.; Jena, K. C.; Lütgebaucks, C.; Okur, H. I.; Roke, S. Three dimensional nano "Langmuir trough" for lipid studies. *Nano Lett.* **2015**, *15*, 5558-5563.

(31) Chen, Y.; Okur, H. I.; Lütgebaucks, C.; Roke, S. Zwitterionic and charged lipids form remarkably different structures on nanoscale oil droplets in aqueous solution. *Langmuir* **2018**, *34*, 1042–105.

(32) Okur, H. I.; Chen, Y.; Smolentsev, N.; Zdrali, E.; Roke, S. Interfacial structure and hydration of 3D lipid monolayers in aqueous solution. *J. Phys. Chem. B* **2017**, *121*, 2808-2813.
